# A simplified, robust protocol for [^18^F]fluoride elution under mild conditions to enhance late-stage aromatic radiofluorinations

**DOI:** 10.1038/s41598-025-27696-1

**Published:** 2025-12-01

**Authors:** Stefan Milton, Alexandros Pappas, Niki Constantinou, Staffan Holmin, Jeroen A. C. M. Goos

**Affiliations:** 1 Clinical Neuroscience, Karolinska Institute, Visionsgatan 4, Stockholm, 17164 Sweden; 2 Neuroradiology, Karolinska University Hospital, Stockholm, Sweden; 3 MedTechLabs, Stockholm, Sweden

**Keywords:** 18F, Radiofluorination, Base, Trapping, Elution, Tetrazine, Chemical biology, Chemistry

## Abstract

Direct radiofluorination of base-sensitive PET precursors is challenging due to the harsh reaction conditions of traditional fluorine-18 radiochemistry. In particular, the presence of inorganic bases with a relatively high conjugate p*K*_a_ (e.g. K_2_CO_3_) and phase-transfer catalysts (e.g. Kryptofix 2.2.2), and/or the need for azeotropic removal of elution solvents during fluorine-18 workup have been detrimental to reaction yields. This has limited the practical applicability of base-sensitive but clinically interesting radiotracers and, ultimately, their accessibility for patient care. By lowering alkalinity and eliminating the need for phase-transfer catalysts and azeotropic drying, we speculated that radiochemical conversions (RCCs) and yields (RCYs) would substantially be improved. We demonstrate that this can be achieved by using a weak anion exchange cartridge for trapping fluorine-18. No preconditioning is required, and fluorine-18 is easily eluted directly into the reaction vessel using a solution of weak base and organic solvent, the types of which can be adjusted to the specific requirements of the desired reaction. We demonstrate the general applicability of the procedure using various elution solutions and base-sensitive, copper-mediated radiofluorinations. As proof of concept, we markedly improve RCCs and RCYs for base-labile tetrazine precursors. To demonstrate its versatility, we expand the procedure to other types of radiofluorination reactions, including non-metal-mediated and isotopic exchange reactions.

**Supplementary Information:**

The online version contains supplementary material available at 10.1038/s41598-025-27696-1.

## Introduction

In positron emission tomography (PET) imaging, a bioactive molecule (i.e. radiotracer) is labelled with a radionuclide and administered to a patient or animal for functional imaging of pathophysiology or other biological processes. The radiotracer emits positrons that upon so-called annihilation with electrons in the surrounding tissue generate two gamma photons of identical energy (511 keV) but opposite direction, which are detected by the PET scanner. Continuous detection of incoming photons during the scan period and their tomographical reconstruction into three-dimensional images provides functional information about biochemical and metabolic processes within the body. Fluorine-18 (^18^F) is currently considered the ideal radionuclide for (pre)clinical PET due to its clinically feasible half-life (109.8 min), low positron energy (max. 0.635 MeV) and clean decay profile (97% positron emission)^1^.

Generally, [^18^F]fluoride is provided as a solution in [^18^O]H_2_O after its production in a cyclotron via the ^18^O(p, n)^18^F nuclear reaction. As a result, [^18^F]fluoride is delivered in strongly hydrated form, which leads to its reduced nucleophilicity and, as such, significant deactivation for essentially nucleophilic radiofluorination reactions. To remove water and impurities, which can both severely compromise the nucleophilic radiofluorinations, [^18^F]fluoride is typically first trapped on a strong anion exchange (SAX) cartridge (e.g. QMA). The trapped [^18^F]fluoride is then eluted into a reaction vessel using an aqueous base (e.g. K_2_CO_3_), often with the addition of acetonitrile and a metal-chelating cryptand such as Kryptofix 2.2.2 (K_2.2.2_) as phase-transfer catalyst to reactivate the [^18^F]fluoride. In the final step, all solvents are removed by azeotropic drying, resulting in an [^18^F]fluoride salt suitable for radiofluorination reactions.

Although this procedure has been established for decades and has proven to be effective for the nucleophilic radiofluorination of a large number of tracers, it carries notable intrinsic constraints. First, inorganic bases with a relatively high conjugate p*K*_a_ (i.e. relatively ‘strong’ bases) are generally required to elute [^18^F]fluoride from the SAX cartridge. This creates an alkaline reaction environment that hampers the radiofluorination of base-sensitive compounds^2^. The presence of such bases could lead to side reactions that result in the hydrolysis, elimination and/or degradation of precursors, intermediates and final products^3–5^. Moreover, next-generation ^18^F-fluorination methods mediated by transition metals (e.g. Cu, Ni, Pd) are holding great promise, but their sensitivity to basic conditions has limited their radiochemical yields^2,3^. Second, it has been indicated that the presence of cryptands like K_2.2.2_ could negatively affect post-fluorination reactions and/or protecting group manipulations^3^. In addition, K_2.2.2_ in particular has considerable acute toxicity, necessitating careful quantification and elimination from radiopharmaceutical preparations before clinical use^6^. Third, the azeotropic removal of water and evaporation of other solvents can be unnecessarily time-consuming (7–30 min) and may lead to a loss of radioactivity of up to 30–50% due to decay and adsorption of [^18^F]fluoride onto the surface of reaction vessels^5,7,8^. Furthermore, this process has proven to be difficult to miniaturise in, for example, microfluidic devices^7,9^.

Numerous alternative procedures have been developed to overcome (part of) these limitations, including the addition of different bases (or their exclusion altogether), salts, neutral onium salts, transition-metal complexes or other additives, the modification of preconditioning and elution protocols or the anion exchange resins themselves, and even the design of sophisticated precursors that allow their radiofluorination in aqueous reaction environments^3,5,7–25^. In the current study, we explore the applicability and versatility of weak anionic exchange (WAX) cartridges for trapping [^18^F]fluoride. By its elution using organic solvents containing readily available non-ionic weak bases, we create a mildly basic reaction environment suitable for base-sensitive radiofluorination reactions and precursors. First, we test the procedure in copper-mediated aromatic radiofluorination reactions, which are known to be base-sensitive. Methods developed by the Sanford, Scott and Gouverneur groups have allowed the nucleophilic copper-mediated radiofluorination of arenes from their trifluoroborate, boronic acid, boronic ester or stannane precursors^26–30^. Despite the sensitivity of these reactions to bases, still K_2_CO_3_ was needed to elute [^18^F]fluoride from the SAX cartridge, contributing to the formation of unproductive copper adducts and potentially damaging base-sensitive precursors^3^. Moreover, the use of aqueous elution solutions required an azeotropic drying step. By using a WAX cartridge, the Krasikova group successfully removed the requirement for aqueous K_2_CO_3_ and subsequent azeotropic drying, but still a relatively large amount (25 µmol) of home-made phase-transfer catalyst (i.e. pyridinium sulphonate salt) was used for elution^23^.

**Fig. 1:**
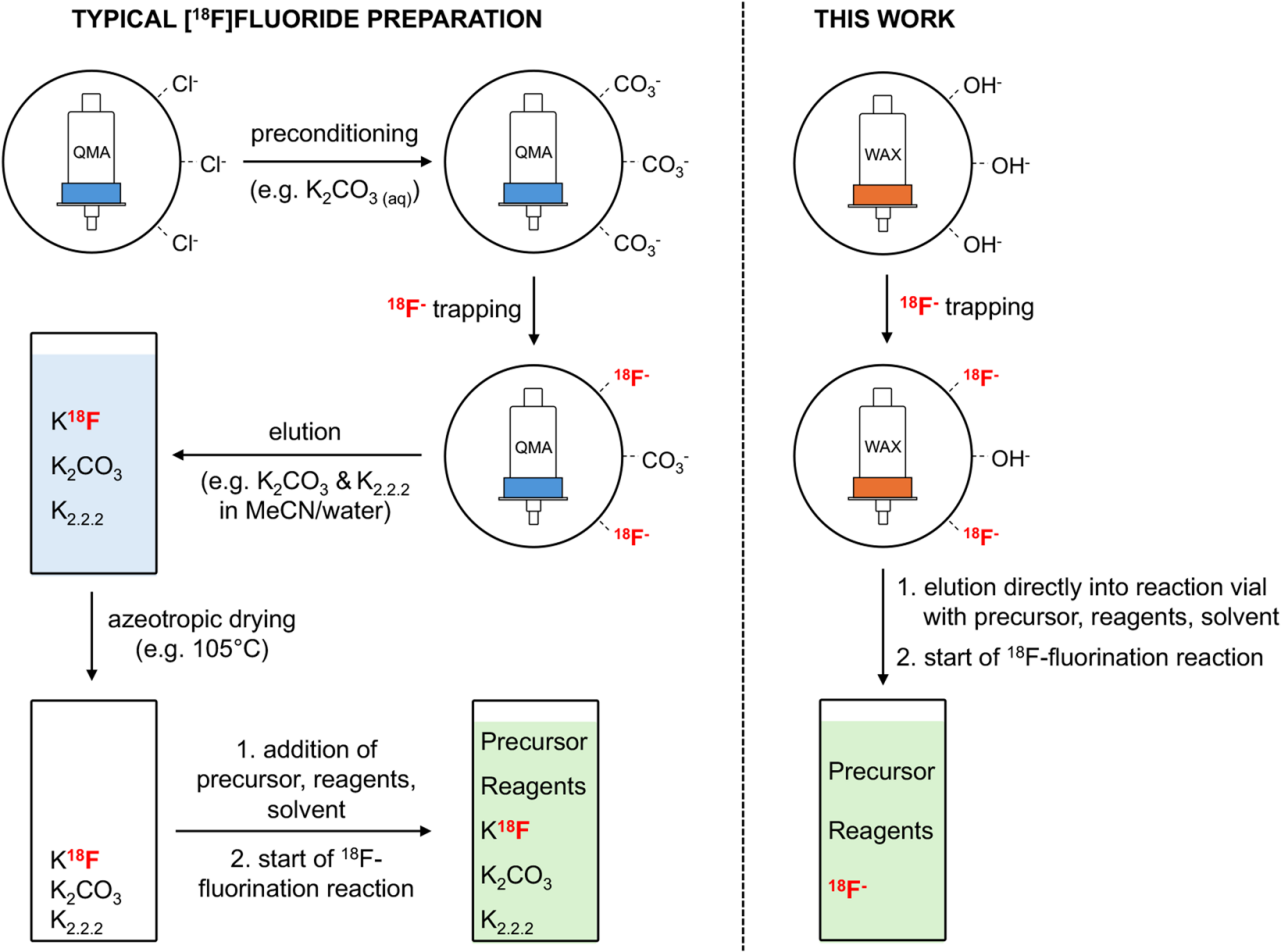
Typical preparation of [^18^F]fluoride for ^18^F-fluorination reactions (left) compared to the method described here (right). QMA: quaternary methyl ammonium, WAX: weak anion exchange. Counter-ions are indicated using dotted lines.

**Fig. 2:**
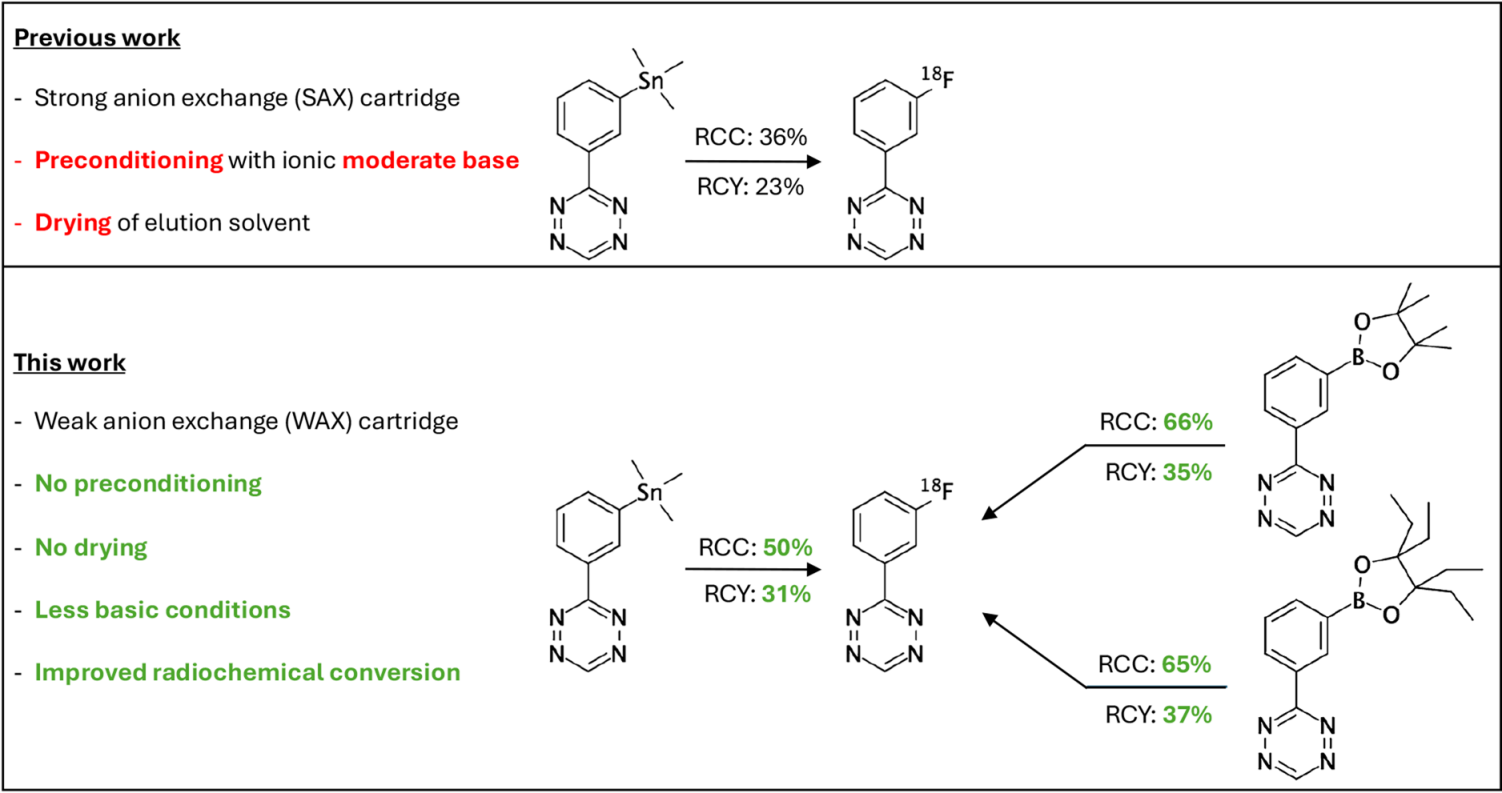
Improvement of ^18^F-fluorination conversions and yields compared to previous attempts^31,32^.

Building on this concept, we aimed to eliminate the need for additives altogether (i.e. preconditioning anions, phase-transfer catalysts) and simplify the trapping and elution procedure of [^18^F]fluoride in general by avoiding preconditioning and drying steps (Fig. 1). We explore various elution conditions and use the otherwise base-sensitive copper-mediated radiofluorination of a commercially available aryl boronic pinacol ester as a model reaction to optimise the procedure. We test the robustness of the procedure by radiofluorinating a number of aryl precursors with different leaving groups and aromatic directing groups to, ultimately, provide proof of concept by radiofluorinating base-labile tetrazine precursors (Fig. 2). The versatility and general applicability of the procedure are emphasised by successfully performing other types of ^18^F-fluorination reactions, including non-catalysed reactions under stronger basic conditions, as well as halogen-^18^F exchange reactions.

**Fig. 3:**
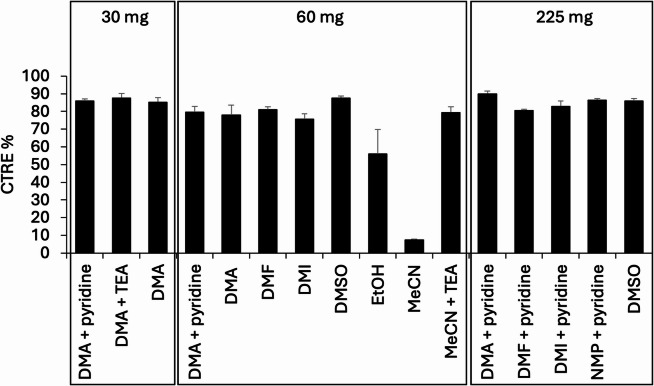
Combined trapping and release efficiency (CTRE) for different elution solutions (3% base), using 30, 60 or 225 mg WAX columns.

## Results

### Evaluation of elution conditions

First, we evaluated the effect of different elution solutions on the combined trapping and release efficiency (CTRE) of [^18^F]fluoride from the WAX cartridge (Fig. 3, Supplementary Figure S1, Supplementary Tables S1–S2). To keep the WAX cartridge procedure as simple and easy-to-perform as possible, we tested the CTRE for different elution solutions without preconditioning the cartridges and without adding any cryptands or other types of phase transfer catalysts. As a starting point, *N*,*N*-dimethylacetamide (DMA) containing 3% pyridine was used as elution solvent. That is, amide-based solvents such as DMA and DMF (i.e. *N*,*N*-dimethylformamide) are generally considered the highest yielding solvents for copper-mediated aromatic radiofluorinations^27–29,33^. Moreover, for these type of radiofluorinations, it has been suggested that an excess of 9–125 equivalents of pyridine results in the highest radiochemical conversions^27–29,33^. In a total reaction volume of 0.5 mL, as is used in this study, 3% of pyridine corresponds to 9–85 equivalents relative to the added amounts of precursor. With this elution solution, the average CTREs were 86% (range: 85–87%) for the 30 mg WAX cartridge, 80% (range: 76–82%) for the 60 mg cartridge and 90% (range: 89–91%) for the 225 mg cartridge, respectively. Interestingly, when using DMA as elution solvent, the added base seemed to hardly affect the CTRE, as the CTRE (30 mg cartridge) was 88% (range: 85–90) when the base was changed to 3% triethylamine (TEA) and still even 85% (range: 83–88%) when no base was added at all. Similarly, changing to other amide-based solvents such as DMF (81%; range: 80–81%), 1,3-dimethyl-2-imidazolidinone (DMI) (83%; range: 81–85%) or *N*-methyl-2-pyrrolidone (NMP) (87%; range: 86–87%), containing 3% pyridine, did not substantially change the CTRE (225 mg cartridge).

To determine the versatility of the WAX columns for their use in other types of radiofluorination reactions (i.e. not copper-mediated), we compared the CTRE with other elution solvents commonly used in radiofluorination reactions, without the addition of any base. Elution (60 mg cartridge) was most efficient for dimethyl sulfoxide (DMSO) (88%; range: 87–89%), DMF (81%; range: 80–83%), DMA (78%; range: 72–83) and DMI (76%; range: 74–79), followed by ethanol (EtOH) (56%; range: 44–71%). Interestingly, the CTRE for acetonitrile (MeCN) was only 7% (range: 7–8%) when no base was added, but when the common (radiochemistry) base TEA was added, the CTRE increased to 79% (range: 77–83%).

Altogether, these data demonstrate that the solutions for eluting [^18^F]fluoride from WAX cartridges could be tailored to the type of radiofluorination reaction performed, without the need for preconditioning anions or other additives.

### Optimisation of conditions for copper-mediated ^18^F-fluorination

**Fig. 4:**
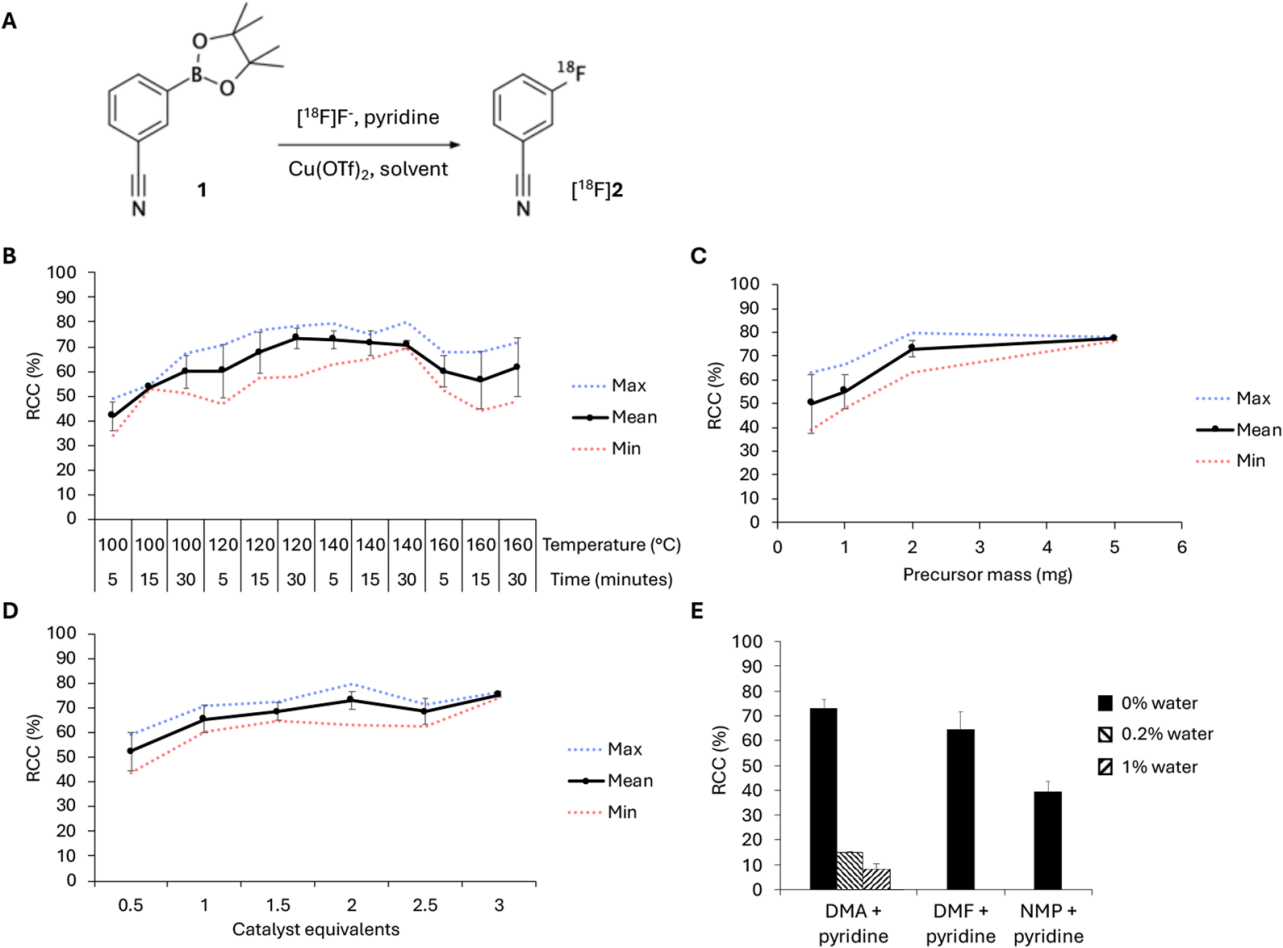
Optimisation of the reaction conditions for (**A**) the model reaction. Optimised reaction conditions include (**B**) reaction temperature and time (using 2 mg precursor, 2 eq. Cu(OTf)_2_ and 3% pyridine in DMA), (**C**) added amount of precursor (using 2 eq. Cu(OTf)_2_ and 3% pyridine in DMA, for 5 min at 140 °C), (**D**) added amount of catalyst (using 2 mg precursor and 3% pyridine in DMA, for 5 min at 140 °C) and (**E**) reaction solvent (using 2 mg precursor, 2 eq. Cu(OTf)_2_ and 3% pyridine, for 5 min at 140 °C) (*n* ≥ 3 for all conditions).

The elution solutions containing [^18^F]fluoride were directly used in subsequent ^18^F-fluorinations, without the need for any drying steps. Conditions were optimised for the copper-mediated ^18^F-fluorination of 3-(4,4,5,5‐tetramethyl‐1,3,2‐dioxaborolan‐2‐yl)benzonitrile (**1**) as model substrate (Fig. 4A). Copper(II) triflate (Cu(OTf)_2_) was used as the copper source due to its low cost and high availability^28^. In each reaction, 3% of pyridine was added following the rationale as described above.

First, the effects of reaction temperature and time on radiochemical conversion (RCC) were measured. Increasing the reaction temperature from 100 °C to 140 °C led to a steadily increasing RCC with a maximum of 80% already after 5 min at 140 °C (Fig. 4B). Further increasing the temperature to 160 °C resulted in a decline of RCC, indicating that the precursor starts to degrade at temperatures higher than 140 °C. At lower temperatures (100–120 °C), the RCC increased with reaction time (5–30 min). Interestingly, however, at 140 °C, longer reaction times did not further improve the RCC.

Next, we measured the impact of precursor amount on the RCC. The highest RCCs were achieved when using 2 mg (9 µmol) precursor or more, with an average RCC of 73% (range: 63–80%) when 2 mg of **1** was added (Table 1, entry #1) and 77% (range: 76–78%) when 5 mg (22 µmol) was added (Fig. 4C). Notably, the reaction could even be performed using minimal precursor amounts while still reaching moderate but acceptable RCCs. That is, an average RCC of 55% (range: 48–67%) was achieved when using 1 mg (4 µmol) precursor, and still 50% (range: 39–63%) conversion was achieved when only using 0.5 mg (2 µmol) precursor.

It has been indicated that the optimal precursor to copper catalyst ratio is 1 equivalent of precursor to 1.5–4 equivalents of copper catalyst^29,31^. Lowering the amount of added Cu(OTf)_2_ to below 1.5 equivalents (69%, range: 65–72%) indeed led to a lower RCC, although RCCs were still acceptable, being 52% (range: 44–59%) when adding 0.5 equivalents and 65% (range: 60–71%) when adding 1 equivalent (Fig. 4D). Increasing the added amount of Cu(OTf)_2_ to more than 2 equivalents (73%, range: 63–80%) did not immensely improve the RCC, as the RCC for 2.5 equivalents of copper catalyst was 69% (range: 63–72%) and 75% (range: 74–76%) for 3 equivalents.

Three amide-based solvents (i.e. DMA, DMF and NMP) were evaluated (Fig. 4E). The highest RCC was achieved when using DMA (73%; range: 63–80%). In DMF, the average RCC was somewhat lower (64%, range: 56–69%), whereas NMP seemed to be a suboptimal solvent for this type of radiofluorination, with an average RCC of 39% (range: 35–44%). To demonstrate the effect of water on the RCC, we deliberately added 0.2–1% of water to the reaction mixture in DMA, which resulted in a significant decline of RCC. This confirms the detrimental effect of water on ^18^F-fluorination efficacy.

In summary, the highest RCC in the model reaction was achieved using the optimised radiofluorination conditions of 2 mg of precursor, 2 equivalents of Cu(OTf)₂ and 3% pyridine in DMA, with a 5-minute reaction time at 140 °C.

**Table 1:**
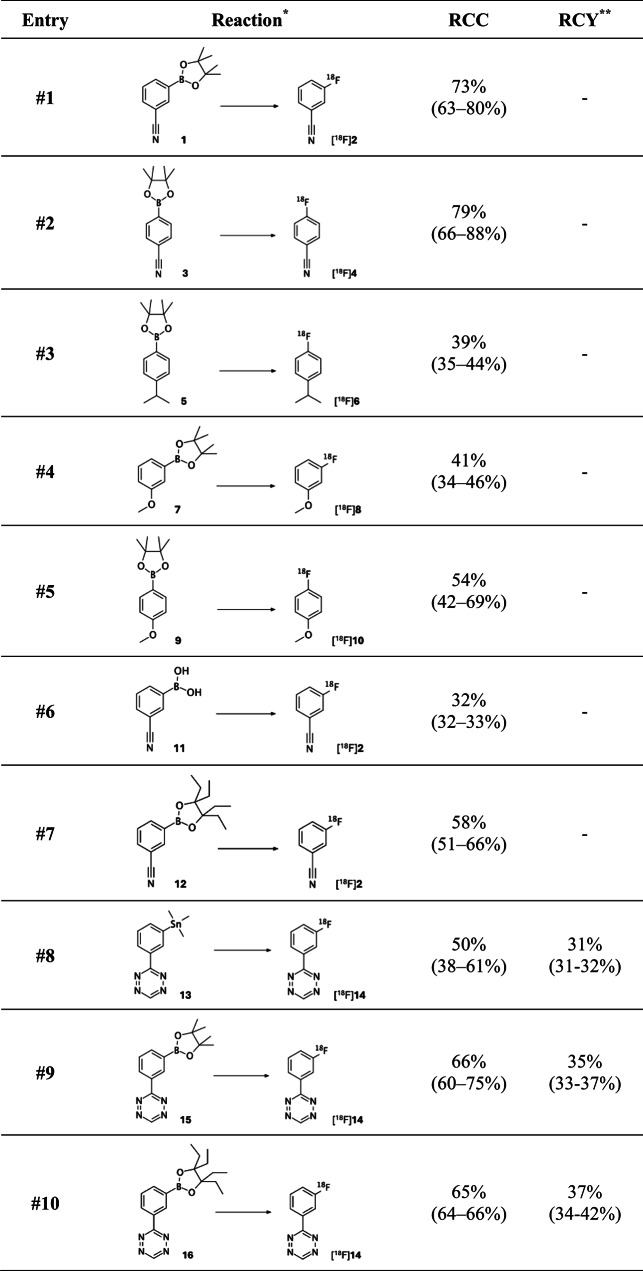
RCC for different precursors radiolabelled under mildly basic, optimal ^18^F-fluorination conditions, including decay-corrected RCY for H-Tzs. ^*^2 Mg Aryl precursor, 2 eq Cu(OTf)_2_, 3% pyridine in DMA, 140 °C, 5 min. ^**^RCY was calculated from and decay-corrected to end-of-bombardment.

### Radiofluorination under mildly basic conditions

To test the robustness of the procedure, we applied the optimised reaction conditions to the ^18^F-fluorination of aryl pinacol (2,3-dimethyl-2,3-butanediol) boronic esters (BPin) with different aromatic directing groups. Overall, precursors containing an aromatic ring activated by electron-withdrawing (nitrile) groups gave higher RCCs, with the highest RCC achieved when the electron-withdrawing group was in the *para* position (79%, range: 66–88%; Table 1, entry #2) compared to the *meta* position (73%, range: 63–80%; Table, entry #1). When the aromatic ring was not activated by electron-withdrawing groups, RCCs were substantially lower. For example, radiofluorination of an aryl precursor with a neutral to slightly electron-donating (isopropyl) group in the *para* position (Table 1, entry #3), the RCC was only 39% (range: 35–44%). Similarly, for aromatic rings containing electron-donating (methoxy) groups, RCCs were only 41% (range: 34–46%) if the electron-donating group was in the *meta* position (Table 1, entry #4) and 54% (range: 42–69%) if the electron-donating group was in the *para* position (Table 1, entry #5).

To further confirm the versatility of the procedure, structurally analogue organoboron precursors with different leaving groups were radiofluorinated. Substituting the BPin in model substrate **1** for a boronic acid (Table 1, entry #6) resulted in a moderate RCC of 32% (range: 32–33%). Substituting the BPin for a 3,4-diethylhexane-3,4-diol boronic ester (BEpin) (Table 1, entry #7) led to a good RCC of 58% (range: 51–66%).

### Radiofluorination of base-sensitive tetrazines

To demonstrate proof of concept, we sought to radiofluorinate base-sensitive precursors that would be of interest to numerous biomedical research groups, including our own. Tetrazines (Tzs) are heterocyclic compounds composed of a six-membered aromatic ring containing four nitrogen atoms. They are extremely reactive toward strained alkenes such as trans-cyclooctene (TCO) in inverse electron demand Diels-Alder (IEDDA) reactions. Their rapid reaction kinetics have made Tzs increasingly popular for (pre)clinical applications, particularly due to their unique suitability for bioorthogonal click chemistry, radiotracer development and nuclear medicine^34–36^. Especially, monounsubstituted 1,2,4,5-tetrazines (H-Tzs) are promising due to their strong in vivo selectivity towards TCO-modified vectors, enabling highly specific pretargeted PET imaging strategies with minimal background signal^34–38^. Unfortunately, the pronounced lability of H-Tzs in traditionally basic ^18^F-fluorination environments has severely limited their wide-spread application and clinical translation. In fact, the maximum reported RCCs for the direct late-stage radiofluorination of H-Tzs do not exceed 36–40%, as has been achieved e.g. for 3-(3‐[^18^F]fluorophenyl)‐1,2,4,5‐tetrazine ([^18^F]**14**), with an average RCY of 23%.^31,32^ We hypothesised that the base-mild procedure described in this communication would benefit the integrity and reactivity of the tetrazine scaffold and, as a result, lead to improved RCCs and RCYs.

Using the optimised procedure, we were able to get notably improved RCCs and RCYs for the radiosynthesis of H-Tz [^18^F]**14**. Starting from the previously reported stannane precursor **13** (Table 1, entry #8), we achieved an average RCC of 50% with a maximum of 61% (range: 38–61%).^31,32^ The average decay-corrected RCY was 31% (range: 31–32%), with a radiochemical purity (RCP) of > 99%. Impressively, however, when using BPin precursor **15** (Table 1, entry #9), we were able to achieve an average RCC of 66%, while in some instances even achieving an RCC as high as 75% (range: 60–75%), with an average decay-corrected RCY of 35% (range: 33–37%) and an RCP of > 99%. Similar results were obtained for BEpin precursor **16** (Table 1, entry #10), with an average RCC of 65% (range: 64–66%), an average decay-corrected RCY of 37% (range: 34–42%) and an RCP of > 99%. To demonstrate that the procedure also supports high-activity reactions, we performed patient-dose radiofluorinations of BEpin precursor **16**, achieving an average molar activity (A_m_) of 20.6 MBq/µmol (range: 20.2–21.4 MBq/µmol).

### Radiofluorination under stronger basic conditions

To further test the robustness and – importantly – broader applicability of the procedure, we aimed to explore whether this simplified approach could also be extended to non-catalysed ^18^F-fluorination reactions that typically require slightly stronger basic conditions. To this end, [^18^F]fluoride was eluted with pure DMSO directly into a reaction vessel containing precursor and tetrabutylammonium bicarbonate (TBAHCO_3_). TBAHCO_3_ is an organic-soluble salt that is approximately 10^5^ times more basic than pyridine and is employed as a base and phase-transfer catalyst in aliphatic and aromatic nucleophilic substitution reactions^10^. It is frequently used in clinical productions, including the synthesis of ^18^F-labelled PSMA from its trimethylammonium precursor^39^. Applying these conditions to benzonitrile precursor **17**, which also contains trimethylammonium as the leaving group (Table 2, entry #1), we achieved a high RCC of 81% (range: 66–86%). Notably, the procedure also translated effectively to halogen exchange (HalEx) reactions (Table 2, entries #2–4). The highest RCC of 80% (range: 78–83%) was observed for the isotopic exchange of ^19^F to ^18^F in the *para* position in precursor **4**. In addition, moderate RCCs were obtained for the isotopic exchange of ^19^F to ^18^F in the *meta* position (52%; range: 45–57%) in precursor **2** and even for the more challenging Br-to-^18^F substitution (41%; range: 21–69%) in precursor **18**. Collectively, this underscores the versatility and adaptability of the procedure across a range of substitution patterns and leaving groups.

**Table 2:**
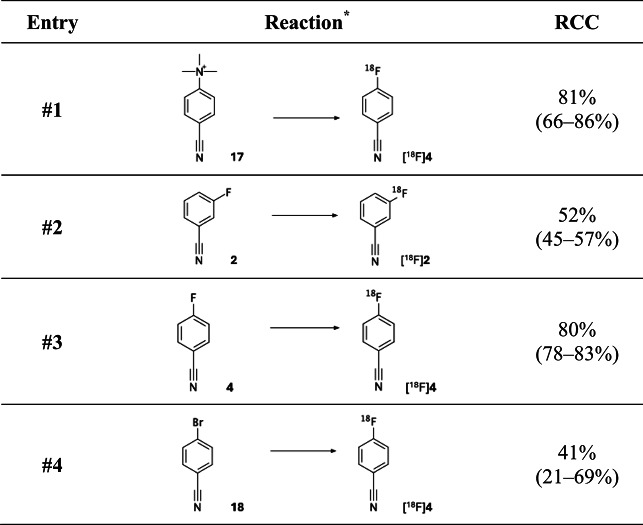
RCC for different precursors radiolabelled under stronger basic ^18^F-fluorination conditions. ^*^2 Mg Aryl precursor, 1 eq TBAHCO_3_ in DMSO, 140 °C, 30 min.

## Discussion

We have developed a simple and effective procedure that is generally applicable to base-sensitive ^18^F-fluorination reactions and substrates, but which is easily adjusted to common ^18^F-fluorination reactions requiring stronger basic conditions. As a proof of concept, we applied the procedure to the direct late-stage radiofluorination of base-sensitive H-Tzs from stannane or boronic ester precursors and were able to achieve conversions up to 75%. As such, we are for the first time reporting a conversion of more than 36–40% for directly radiofluorinated H-Tzs^31,32^. Consequently, this led to substantially improved RCYs for the isolated H-Tzs. In fact, this study reports on the first direct late-stage radiofluorination of H-Tzs from boronic ester precursors (**15–16**). In general, aryl stannane precursors are lower yielding in copper-mediated radiofluorinations compared to aryl boronic precursors, due to their greater susceptibility to protodestannylation under acidic and basic conditions. Similarly, boronic acids are more prone to protodeboronation than boronic esters, which may negatively affect the overall conversion of the radiofluorination reaction, albeit highly depending on the precursor^40^. Recently, 3,4-diethylhexane-3,4-diol boronic ester (BEpin) precursors have emerged as an attractive alternative to traditional Bpin precursors. That is, they retain the structural and electronic characteristics required for successful radiofluorination reactions, while offering superior stability against protodeboronation, along with improved chromatographic behaviour which facilitates purification and leads to higher synthetic yields of precursors^30,41,42^. By using boronic ester precursors, we were able to obtain 1.5-to-2-fold higher RCCs and RCYs than what had been achieved previously^31,32^. Moreover, the procedure allowed for the production of radiofluorinated H-Tzs with a molar activity that corresponds to a typical patient dose of approximately 200 MBq. Although previous automated approaches have achieved higher molar activities by starting with higher initial activity^31^, we refrained from using high amounts of activity due to radiation exposure limits and maximum allowable hand dose associated with manual labelling. In addition to the use of boronic ester precursors, the strongly reduced alkalinity in combination with the exclusion of unnecessary additives contributed to the higher conversions and yields achieved in this study for the H-Tzs, as well as for most other tested precursors^2,10^.

Several alternative approaches have been reported for the radiolabelling of base-sensitive precursors. To limit basicity, it has been suggested to decrease base concentrations^43^, exclude bases altogether^7,14^ or replace traditional inorganic bases by milder and/or organic bases^3,5,9^, salts^5,15,42,44,45^, neutral onium salts^11,16–19^ or transition-metal complexes^20,21^. An added benefit is that in many of these cases, the suggested methodology supplanted the need for azeotropic drying. Impressive results were obtained with these methods, although several procedures still relied on specific additives, preconditioning steps or custom reagents, which may hamper their general applicability and clinical translation. In contrast, the procedure described in this study simplifies the entire [^18^F]fluoride preparation workflow by removing the need for preconditioning, drying, cryptands or phase-transfer catalysts, and instead utilises commercially available weak bases and solvents easily tailored to the reaction type. Overall, this results in a broadly applicable workflow that avoids additives, accommodates base-sensitive substrates and can be adapted for both copper-mediated and non-catalysed fluorination reactions.

The Krasikova group first reported the use of WAX cartridges for trapping [^18^F]fluoride, and the elimination of the azeotropic drying step combined with the reduced base content led to high RCCs for a small set of simple aryl boronic esters^23^. Here, we expanded on the success of that study and further refined and simplified the procedure in order to be applicable to the ^18^F-fluorination of base-sensitive substrates, such as H-Tzs. Importantly, we demonstrated that neither preconditioning nor the addition of phase-transfer catalysts is required for eluting [^18^F]fluoride from the WAX cartridge or for the subsequent copper-mediated ^18^F-fluorination. As a result, potential interference from unnecessary additives is minimised, and their reduced use is likely to facilitate purification and work-up procedures in clinical productions. Moreover, we demonstrate that high RCCs could also be achieved in shorter reaction times at higher temperatures. The successful direct radiolabelling of H-Tzs at higher temperatures has so far been elusive due to the combination of high temperatures with strong bases in traditional [^18^F]fluoride preparation procedures that could lead to decomposition of the base-sensitive H-Tzs^31,32^. The mild alkalinity of the elution/reaction solution in the procedure described here eradicates this issue. Furthermore, in contrast to the optimised radiofluorination procedure of this study, previous H-Tz radiofluorination protocols have included preconditioning and drying steps, which could both lead to lower yields^5,31,32^. As such, the current study provides a simplified and robust procedure for the easy-to-perform^18^F-fluorination of H-Tzs with high radiochemical conversion.

One of the strong advantages of the procedure is that [^18^F]fluoride could be eluted directly into a reaction vial containing all precursors and reagents needed for subsequent radiofluorination reactions, without the need for any solvent evaporation. Various common solvents for radiofluorination reactions (i.e. DMA, DMF, DMSO, DMI, EtOH, MeCN, NMP) could be used to elute [^18^F]fluoride from the WAX cartridge, mostly without the absolute need for any additives to release the [^18^F]fluoride. The high CTRE that we observe here, without the need for preconditioning, is attributed to the hydrophilic-lipophilic balanced (HLB) copolymer backbone of the WAX sorbent, which is composed of *N*-vinylpyrrolidone and divinylbenzene, combined with piperazine moieties that can electrostatically interact with anionic species such as fluoride (Supplementary Figure S1). Unlike strong anion exchangers, these weakly basic sites do not require preconditioning with counterions to initiate ion exchange. The water-wettable nature of the HLB matrix ensures that the sorbent remains active even after drying, allowing efficient trapping of [^18^F]fluoride directly from aqueous solutions. Moreover, the absence of competing counterions may actually enhance fluoride retention, while the pore structure and surface chemistry facilitate efficient elution across a range of solvents. In some cases (e.g. MeCN), the addition of base to the elution solution could substantially enhance the CTRE. These data illustrate the versatility of the procedure, which could easily be adapted to precursors and reagents with differing solubility and to the type of radiofluorination reaction at hand.

In all radiofluorination reactions tested here, 3% of pyridine was added as a base since its importance in copper-mediated radiofluorinations has been demonstrated in numerous studies^23,28,29^. Although other forms of pyridine, for example as pyridinium sulphonate or in combination with a copper source like tetrakis(pyridine)copper(II) triflate (Cu(OTf)_2_(py)_4_), have been used as replacement or in addition to pyridine, we opted for pure pyridine due to its easy accessibility and low cost^23,28,29^. Moreover, by using pyridine in its simplest form, we keep the amounts of unnecessary counterions or additives in the reaction mixture to a minimum, which should facilitate purification of the final product.

Even though the optimised reaction time and temperature for the substrates tested here are 5 min and 140 °C, respectively, it is worth mentioning that equally high RCCs could be achieved at lower temperatures with longer reaction times. That is, reacting for 30 min at 120 °C or even at 100 °C still leads to high-to-acceptable RCCs. For precursors that are sensitive to heat, performing the radiofluorination at lower temperatures using the simplified procedure described here could still lead to high yields.

Similarly, precursor and catalyst amounts may need to be adjusted to each precursor separately to achieve maximal RCC. We aimed to perform the ^18^F-fluorinations with significantly lower precursor amounts than for the majority of previous studies, where the use of up to 60 µmol of precursor has been reported^7,12,23,30^. Although higher RCCs were indeed obtained with higher precursor amounts, we confirm that acceptable RCCs are still achieved with precursor amounts as low as 2 µmol, in line with previous work with the WAX cartridge^23^.

Likewise, the type of catalyst and solvent may need to be catered to the to-be-labelled precursor and reaction constituents. We selected Cu(OTf)_2_ as the readily available and inexpensive copper source, which in combination with pyridine is suggested to promote copper-mediated radiofluorination reactions as effectively as the more costly Cu(OTf)_2_(py)_4_^28^. On the other hand, it has been reported that the use of Cu(OTf)_2_(py)_4_ could lead to improved RCCs for some reactions^27,31,46^. As such, the use of Cu(OTf)_2_(py)_4_ may improve the RCC for some of the precursors tested here. Similarly, it has been demonstrated that the addition of alcohols to the reaction mixture could enhance the yield of copper-mediated radiofluorinations due to the decreased basicity of alcohol-dissolved fluorine without compromising its nucleophilicity^47,48^. These potential improvements will be explored in follow-up studies. DMA as radiofluorination solvent resulted in the highest RCC for the model reaction, which is consistent with previous studies that indicate that DMA leads to higher RCCs for electron-deficient arenes compared to, for example, DMF^33^. Nevertheless, good yields in copper-mediated radiofluorinations have also been achieved with other amide-based solvents such as DMF, DMI and NMP^27–30,33^. The presence of water in the reaction mixture had a strong negative effect on the RCC. This could be explained by the high moisture-sensitivity of copper catalysts, as coordination with water can deactivate the catalytic species, impair oxidative addition or disrupt the formation of reactive copper intermediates^49^. It also indicates that the majority of residual water could indeed simply be removed by washing the WAX cartridge with acetonitrile before elution, confirming that additional drying procedures are not required. All in all, the optimised reaction conditions established here agree with previously described preferred conditions for copper-mediated radiofluorinations^29^.

Applying the optimised reaction conditions to the ^18^F-fluorination of different substrates with varying aromatic directing groups resulted in high RCCs in particular for electron-deficient arenes in which the phenyl ring was activated by an electron-withdrawing group in the *para* position. Activation of the phenyl ring is a requirement for effective ^18^F nucleophilic aromatic substitutions (S_N_Ar), which benefit from electron-withdrawing groups located in the *para* position^50^. In fact, we obtained higher RCCs for [^18^F]**2** and [^18^F]**4** compared with most previous publications using the same BPin precursors, which reported RCCs of 11–59% and 39–73%, respectively^27,46,51–54^. Notably, only one recent study achieved a substantially increased RCC for [^18^F]**4**, from 43% to 97%, by changing the reaction solvent from DMF to DMI^30^. This approach should be considered for future optimisation. Electron-donating groups such as alkyl or methoxy groups increase the electron density of the aromatic ring, which generally hinders S_N_Ar reactions. Although RCCs for those substrates were indeed lower, they were still well within the acceptable range for ^18^F-fluorinations in general. Moreover, for [^18^F]**10**, the RCC was in the same range as previous reports (4–71%) using the same precursor^43,51,54,55^, although higher RCCs could be achieved (86–89%) when a phase-transfer catalyst was added (*note*: besides the use of a phase-transfer catalyst and slightly different reaction conditions, the highest RCC of 89% was reported using a similar WAX-based procedure)^23,56^. Similarly, achieving higher RCCs for any of the other substrates tested in this study could likely be achieved using the presented procedure by tailoring the reaction conditions to each substrate separately, for example by adjusting reaction times and temperatures, types of catalyst or reaction solvents, or by the addition of, for example, phase-transfer catalysts.

In conclusion, this study presents a simple and robust procedure for ^18^F-fluorinations under mildly basic conditions. Using a weak anion exchange (WAX) resin, [^18^F]fluoride is efficiently trapped and eluted directly into a base-mild organic reaction environment, suited for base-sensitive ^18^F-fluorination reactions and substrates. The method eliminates the need for preconditioning, additives or solvent evaporation, thereby simplifying the standard preparation of [^18^F]fluoride for nucleophilic substitutions. We demonstrated its versatility by successfully applying the procedure to ^18^F-fluorination reactions requiring stronger alkaline conditions, including copper-free aromatic substitution and halogen exchange, with RCCs comparable to previously reported values^9,57–61^. Notably, the successful Br-to-^18^F halogen exchange reaction with fairly high RCCs is particularly significant, given the widespread availability and straightforward synthesis of brominated aromatic precursors relevant to nuclear medicine. This enhances the practical utility of the method for late-stage radiolabelling of diverse PET tracers. Overall, this approach offers a broadly applicable, robust and mild protocol for ^18^F-fluorination reactions in general.

## Methods

Detailed experimental procedures as well as additional analytical data are available in the Supplementary Information.

### Evaluation of elution conditions

Non-carrier-added [^18^F]fluoride (0.5–3 GBq, 0.5–2 mL) was trapped on 30 mg, 60 mg or 225 mg Oasis^®^ WAX cartridges (prewetted with 5 mL water) without the need to introduce any preconditioning anions or additives. To remove residual water, the cartridge was purged with air, followed by a wash using 5 mL MeCN and another purge. For the 225 mg cartridges, [^18^F]fluoride was loaded and washed via the male side. [^18^F]F^−^was eluted slowly using organic solvent (DMA, DMF, DMSO, DMI, EtOH, MeCN, NMP), with or without 3% weak organic base (pyridine, TEA), in 1 mL fractions (5 mL in total) with the majority of activity eluting in the first fraction (Supplementary Table S2). The combined trapping and release efficiency (CTRE) was defined as the ratio of eluted activity relative to the starting activity before trapping. CTRE was reported as the average of *n* ≥ 3 elutions for the 30 mg and 60 mg cartridges and as the average of *n* = 2 elutions for the 225 mg cartridge.

### General ^18^F-fluorination procedure

No-carrier-added [^18^F]fluoride was trapped and eluted as described in the previous section. Without the need for any drying steps, in the optimised procedure, subfractions of 10–100 MBq (for high molar activity reactions: 0.5-1 GBq) were added directly to pre-dried glass reaction vials containing aryl precursor (2.0 mg) and copper(II)triflate (Cu(OTf)_2_) (2 eq.) in a total reaction volume of 500 µL 3% pyridine in DMA. The reaction mixture was stirred in a closed reaction vial for 5 min at 140 °C. For ^18^F-fluorinations under stronger basic conditions, pure DMSO was used as elution and reaction solvent and TBAHCO_3_ (1 eq.) was added instead of copper catalyst, with a reaction time of 30 min. After cooling down the reaction mixture, analysis was performed by iTLC to calculate the RCC and analytical radio-HPLC to confirm successful synthesis of the desired product. Unlabelled ^18^F and other impurities were removed from radiolabelled tetrazine products by quenching the reaction mixture with 20 mL water and trapping the product on a Waters Sep-Pak^®^ C18 Plus Short cartridge (conditioned using 5 mL ethanol and then 10 mL water). The product was eluted slowly in 0.5 mL fractions of ethanol. RCY was decay-corrected and calculated from the starting activity at end-of-bombardment. RCP of the isolated product was confirmed by iTLC. RCC, RCY, RCP and A_m_ were reported as the average of *n* ≥ 3 radiofluorination reactions.

## Supplementary Information

Below is the link to the electronic supplementary material.

Supplementary Material 1

## Data Availability

All data generated or analysed during this study are available from the corresponding author on reasonable request.
