# Supplementary material for: A simplified, robust protocol for [18F]fluoride elution under mild conditions to enhance late-stage aromatic radiofluorinations
Source: Sci Rep. 2025 Dec 1;15:42929. doi: 10.1038/s41598-025-27696-1 (PMC12673099; doi:10.1038/s41598-025-27696-1)
Supplement: Supplementary file 1 — Supplementary Material 1 [file 41598_2025_27696_MOESM1_ESM.docx]

**MATERIALS AND METHODS**

**Materials**

Chemicals were obtained from Merck (Solna, Sweden) and used without further purification, unless specified otherwise. *m*-CN-Ph-BPin (**1**), *p*-CN-Ph-BPin (**3**), iPr-Ph-BPin (**5**), *m*-MeO-Ph-BPin (**7**), *p*-MeO-Ph-BPin (**9**), CN-Ph-B(OH)_2_ (**11**), as well as their corresponding fluorinated reference materials were purchased from Doug Discovery (Walldorf, Germany). Ethanol was purchased from Fisher Scientific (Gothenburg, Sweden). *N,N*-Dimethylacetamide (DMA), *N,N*-dimethylformamide (DMF), dimethyl sulfoxide (DMSO), acetonitrile (MeCN), ethanol (EtOH), *N*-methyl-2-pyrrolidone (NMP), 1,3-dimethyl-2-imidazolidinone (DMI), tetrahydrofuran (THF), pyridine and triethylamine (TEA) were dried over 3 Å molecular sieves before use. THF was also passed over basic aluminium oxide to remove stabilising agent (butylhyrozititulouene, BHT). Tetrabutylammonium bicarbonate (TBAHCO_3_) was obtained by treating a 1/4 (v/v) solution of tetrabutylammonium hydroxide (40% solution in water) in ethanol with an excess of dry ice, followed by lyophilisation and resuspension of the solid salt in dry DMSO to a concentration of 80 mg/ml. Water was distilled and deionised (18.2 MΩ) by an Elga Purelab Flex water purification system (AB Ninolab, Upplands Väsby, Sweden). Oasis® WAX 1 cc Vac (30 mg, 30 µm), WAX 3 cc Vac (60 mg, 60 µm), WAX Plus Short (225 mg, 60 µm) and Sep-Pak^®^ C18 Plus Short (360 mg, 55 - 105 µm) cartridges were purchased from Waters (Solna, Sweden).

**Instrumentation**

Manual column chromatography was performed in glass columns packed with silica gel (60 Å, 40–63 micron; Fisher Scientific, Gothenburg, Sweden) as the stationary phase. Semi-automated flash chromatography was performed on a Pretech Instruments flash system (Sollentuna, Sweden) using silica-packed cartridges (SillaSep™️ flash cartridges, 60 Å, 40–63 micron; SiliCycle, Paris, France).

Thin-layer chromatography (TLC) of non-radioactive products was performed using Macherey-Nagel™ Standard Alugram™ aluminum sheets (Fisher Scientific, Gothenburg, Sweden), pre-coated with silica 60 and a 254 nm fluorescence indicator, using 100% toluene as Mobile Phase A or a mixture of hexane/ethyl acetate = 95/5 (v/v) as Mobile Phase B. Instant thin-layer chromatography (iTLC) of radioactive products was performed using reversed-phase aluminium TLC sheets (Merck, Solna, Sweden), pre-coated with RP-18-modified silica gel and a 254 nm fluorescence indicator, using MeCN/water = 75/25 (v/v) as the mobile phase. For iPr-Ph-BPin (**5**), 100% EtOAc was used as mobile phase. iTLC plates were analysed on a BIOSCAN AR 2000. RCC was defined as area-under-the-curve for product divided by total area-under-the-curve.

^1^H and ^13^C NMR spectra were recorded on a Bruker Ascend 400 MHz NMR spectrometer (9.4T) at room temperature, operating on Bruker TopSpin (400'54 Ascend). Chemical shifts (δ) are reported in ppm and the residual solvent signal is used as the internal calibration standard (CDCl_3_: ^1^H = 7.26 ppm, ^13^C =77.16 ppm; D_2_O: ^1^H = 4.79 ppm). ^13^C NMR spectra were obtained with complete proton decoupling. For precursors Tz-Ph-SnMe_3_ (**13**) and Tz-Ph-BPin (**15**), ^13^C DEPT-135 NMR was performed. Multiplicity is reported as follows: s = singlet, d = doublet, t = triplet q = quartet, m = multiplet, dd = doublet of doublets, ddd = doublet of doublets of doublets, td = triplet of doublets, tdd = triplet of doublets of doublets, brs = broad singlet. The coupling constant *J* is reported in hertz (Hz).

An Agilent 1200 Infinity II system, equipped with a multisampler (G7167A), quaternary pump (G7111A), DAD UV detector (G7115A) and a G6125B MS mass spectrometer, was used for high performance liquid chromatography - mass spectroscopy (HPLC-MS). Within this system, for reverse-phase high-performance liquid chromatography (HPLC), a Waters XBridge C18 column (50 × 3.0 mm, 3.5 µm) was used at 40 °C (MeCN/ NH_4_HCO_3 (aq)_, 5/95 to 60/40 (v/v) in 3 minutes at flow rate 1 mL/min^-1^). For mass spectroscopy (MS), a quadrupole atmospheric pressure ionisation - electrospray (API-ES) source was used with a capillary voltage of 4000 V (positive ion mode) or 3500 V (negative ion mode) and a drying gas temperature of 350 °C. Unless otherwise stated, mass spectra are reported in positive ion mode.

Semi-preparative HPLC was performed on an Atlantis Premier BEH C18 AX column (250 × 4.6 mm 5 µm; Waters, Solna, Sweden) at flow rate 1 mL/min^-1^ and wavelengths 254 nm and 535 nm. Preparative HPLC was performed on a Machery-Nagel VP Nucleosil 100–5 C18 Nautilus column (250 × 10 mm, 5 µm; RAMC AB, Sollentuna, Sweden) at flow rate 5 mL/min^-1^ and wavelengths 254 nm and 535 nm. Method A: MeCN/water, 25/75 to 35/65 (v/v) in 15 minutes. Method B: MeCN/water/TFA, 50/50/0.1 to 95/5/0.1 (v/v/v) in 15 minutes.

**Synthesis of 3‐(3‐iodophenyl)‐1,2,4,5‐tetrazine (Tz-Ph-I) (20)**

**
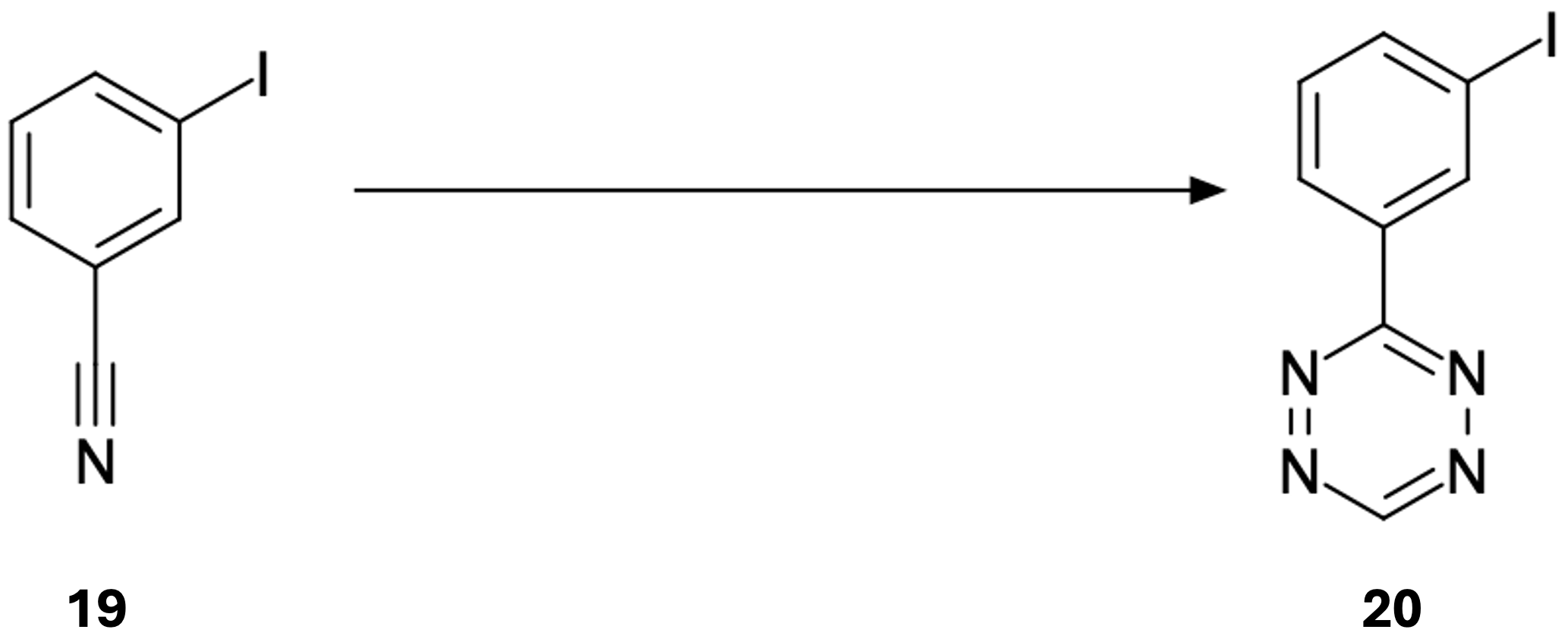
**

Formamidine acetate (4.0 g, 38.4 mmol) and 3-iodobenzonitrile (**19**; 2.22 g, 9.7 mmol) were mixed in 10 mL ethanol (99.8%) in a 15 mL heavy-wall cylindrical Synthware pressure vessel, equipped with a magnetic stirring bar. Hydrazine monohydrate (64–65%; 4.0 mL, 77 mmol) was slowly added in fractions of 1 mL while stirring, after which the reaction mixture was pipetted up and down multiple times. 3-Mercaptopropionic acid (200 µL, 2.26 mmol) was added as catalyst and the pressure vessel was left open for 5 minutes to release any formed gases. The pressure vessel was closed and heated to 55^o^C in a sand bath under magnetic stirring. After 22 hours, the pressure vessel was allowed to cool down to room temperature for 30 minutes and then opened gently to release potential overpressure. The reaction mixture was transferred to a 500 mL conical flask, followed by the addition of 5.34 g (77 mmol) sodium nitrite in 35 mL water and 60 mL dichloromethane (DCM) under magnetic stirring. The crude product was oxidised by slowly adding 140 mL 1 M HCl to reach a pH of 4 (reaction mixture turns bright red). After 10 minutes, the product was extracted 3 times using DCM and water. The organic phase was dried over magnesium sulphate, filtered and concentrated *in vacuo*. Purified Tz-Ph-I (**20**; 1.1 g, 40% yield) was obtained *via* column chromatography (100% toluene) as a red solid. TLC Mobile Phase A was used for qualitative analysis. ^1^H NMR (400 ΜΗz, CDCl_3_) *δ* 10.25 (s, 1H), 9.00 ( t, *J* = 1.72 Hz, 1H), 8.61 (ddd, *J* = 7.90 Hz, 1.62 Hz, 1.10 Hz, 1H,) 8.00 (ddd, *J* = 7.90 Hz, *J* = 1.71 Hz, *J* Ar= 1.1 Hz, 1H), 7.35 (t, *J* = 7.90 Hz, 1H). ^13^C NMR (101 MHz, CDCl_3_), *δ* 165.50 (s, 1C), 158.20 (s, 1C) 142.14 (s, 1C), 137.28 (s, 1C), 133.64 (s, 1C), 131.11 (s, 1C), 127.53 (s, 1C) 95.04 (s, 1C) (Supplementary Figure S2). LC-MS (API-ES) *m/z*: [M + H]^+^ calculated: 284.1; found: 285.

**Synthesis of 3‐(3‐(trimethylstannyl)phenyl)‐1,2,4,5‐tetrazine (Tz-Ph-SnMe_3_) (13)**

**
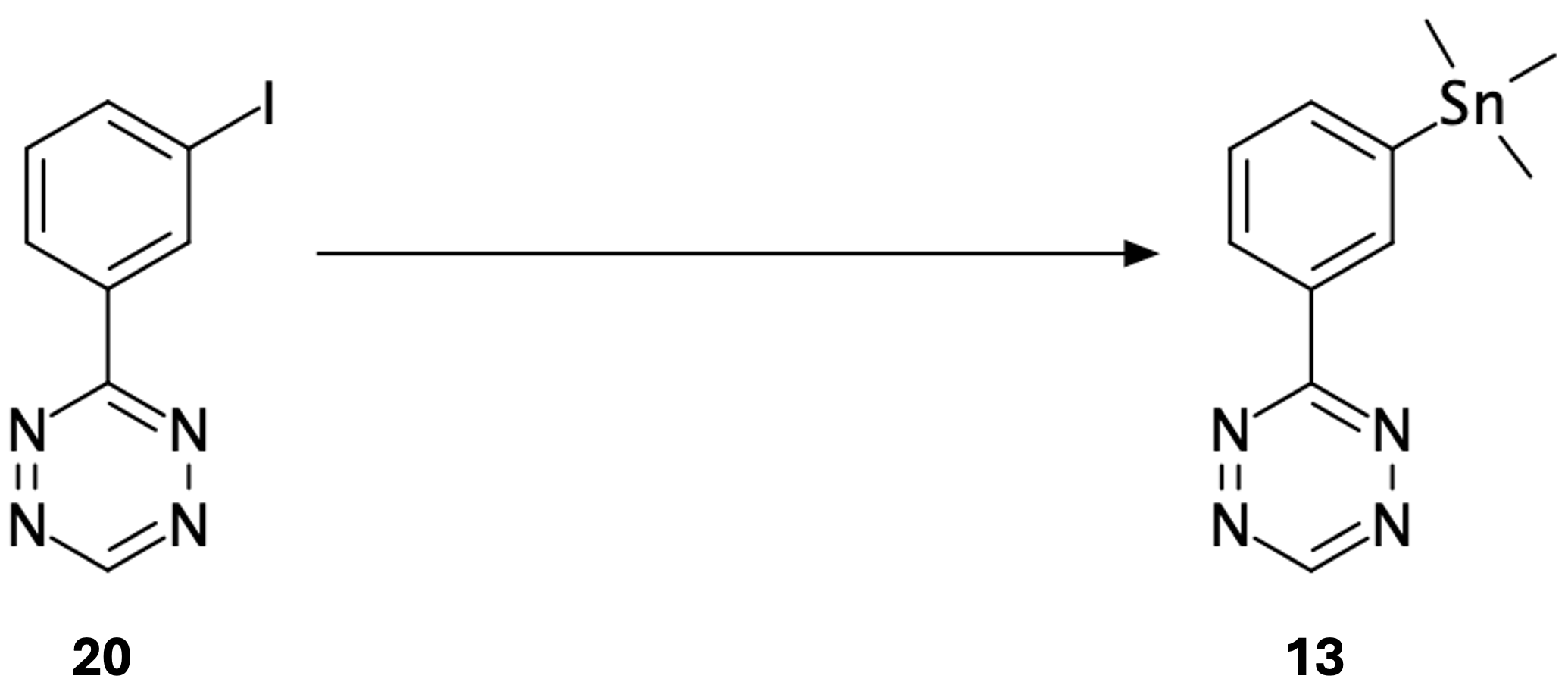
**

All glassware was pre-dried at 105 °C overnight before use and solvents were purged with argon for at least 30 minutes prior to the reaction. Tetrakis(triphenylphosphine)palladium(0) (206 mg, 0.18 mmol) and hexamethylditin (930 µL, 4.5 mmol) were added to a 2-neck round-bottom flask containing a thermometer, after which the flask was purged with argon. THF (7.5 mL) was added to the reaction flask using an argon-purged syringe, followed by the addition of Tz-Ph-I (**20**; 500 mg, 1.8 mmol). Again, THF (7.5 mL) was added to the reaction flask using an argon-purged syringe, after which the flask was sealed with a septum and stirred at 65 °C for 5 hours. To reverse potential reduction of the 1,2,4,5-tetrazine (indicated by a colour change from red to black), the reaction mixture was purged with air for approximately 15 minutes. The mixture was allowed to cool to room temperature before it was quenched with 2.5 mL saturated potassium fluoride and extracted 3 times with DCM and brine. The organic phase was dried over magnesium sulphate, filtered and concentrated *in vacuo*. The crude product was purified by manual column chromatography (hexane/ethyl acetate, 95/5 (v/v)) to afford Tz-Ph-SnMe_3_ (**13**; 98 mg, 17% yield) as a red solid. TLC Mobile Phase B was used for qualitative analysis. ^1^H NMR (400 ΜΗz, CDCl_3_) *δ* 10.21 (s, 1H), 8.81–8.67 (m, 1H), 8.58–8.53 (m,1H), 7.85–7.71 (m, 1H), 7.57 (ddd, *J* = 7.9, 7.1, 0.6 Hz, 1H). ^13^C DEPT-135 NMR (101 MHz, CDCl_3_) *δ* 157.71 (s, 1C), 140.55 (s, 1C), 135.43 (s, 1C), 128.69 (s, 1C), 128.12 (s, 1C), < 0 (s, 3C) (Supplementary Figure S3). LC-MS (API-ES) *m/z*: [M + H]^+^ calculated: 321.0; found: 322.

**Synthesis of 3‐(1,2,4,5‐tetrazin‐3‐yl)phenylboronic acid pinacol ester (Tz-Ph-BPin) (15)**

**
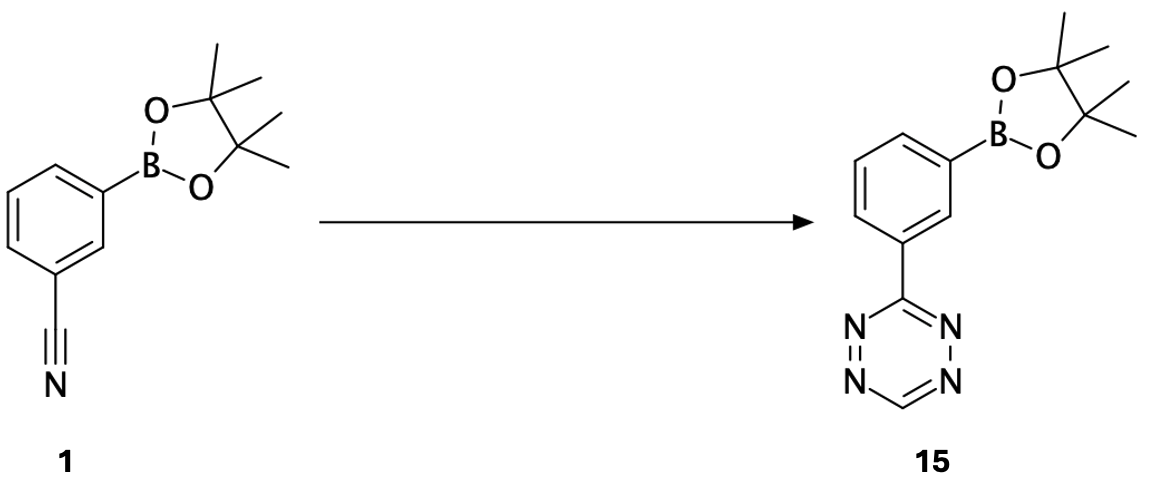
**

Using 3‐(4,4,5,5‐tetramethyl‐1,3,2‐dioxaborolan‐2‐yl)benzonitrile (**1**; 2.22 g, 9.7 mmol) as starting material, the same reaction procedure, including work-up, was followed as described for **20** (Tz-Ph-I). Purification was first performed by manual column chromatography (toluene/ethyl acetate, 6/1 (v/v)), yielding 0.998 g crude product. A 70 mg fraction of the crude product was further purified by preparative HPLC (Method A), affording Tz-Ph-BPin (**15**; t_R_ = 7.1 min; 16 mg, 8% overall yield) as a red solid. TLC Mobile Phase A was used for qualitative analysis. ^1^H NMR (400 ΜΗz, CDCl_3_) *δ* 10.22 (s, 1H), 9.08 (s, 1H), 8.70 (d, *J* = 7.99 Hz, 1H), 8.0 (d, *J* = 7.36 Hz, 1H), 7.62 (t, *J* = 7.50 Hz, 1H), 1.38 (s, 12H). ^13^C DEPT-135 NMR (101 MHz, CDCl_3_) *δ* 157.8 (s, 1C), 139.28 (s, 1C), 134.76 (s, 1C), 130.79 (s, 1C), 128.69 (s, 1C), 24.86 (s, 4C) (Supplementary Figure S4). LC-MS (API-ES) *m/z*: [M + H]^+^ calculated: 284.1; found: 285.

**Synthesis of 3-(4,4,5,5-tetraethyl-1,3,2-dioxaborolan-2-yl)benzonitrile (CN-Ph-BEpin) (12)**

**
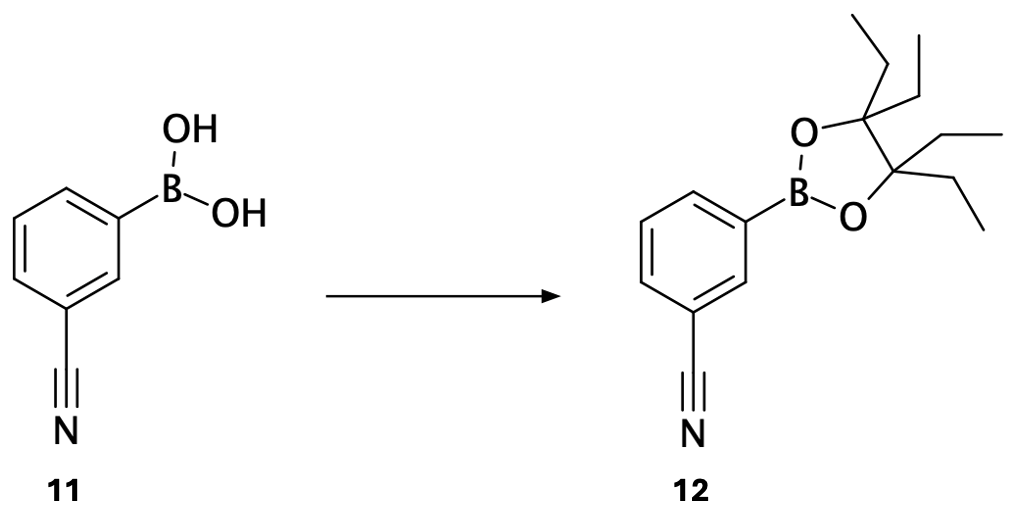
**

3-Cyanophenylboronic acid (**11**; 1 eq, 2.0 g, 13.61 mmol) was added to a solution of 3,4-diethyl-3,4-hexanediol (1.1 eq, 2.82 mL, 14.9 mmol) in 50 mL absolute ethanol. After stirring the mixture at room temperature for 1h, it was concentrated under reduced pressure and redissolved in 40 mL DCM. After again stirring the mixture at room temperature for 1h, it was concentrated under reduced pressure and purified by manual column chromatography (hexane/ethyl acetate, 95/5 (v/v)) to obtain CN-Ph-BEpin (**12**; 2.69 g, 69% yield) as a white solid. TLC Mobile Phase B was used for qualitative analysis. ^1^H NMR (400 ΜΗz, CDCl_3_) *δ* 8.09 (br, 1H), 8.01 (dt, *J* = 7.51, 1.16 Hz, 1H), 7.71 (dt, *J* = 7.8, 1.5 Hz, 1H) 7.46 (t, *J* = 7.7 Hz, 1H) 1.86–1.66 (m, 8H), 0.96 (t, *J* = 7.56 Hz, 12H). ^13^C NMR (101 MHz, CDCl_3_) *δ* 138.90 (s, 1C), 138.54 (s, 1C), 134.35 (s, 1C), 130.68 (brs, 1C), 128.48 (s, 1C) 119.07 (s, 1C), 112.14 (s, 1C), 89.63 (s, 2C), 26.55(s, 4C), 8.92 (s, 4C) (Supplementary Figure S5). LC-MS (API-ES) *m/z*: [M + H_2_O]^+^ calculated: 285.2; found: 303.

**Synthesis of 3-(3-(4,4,5,5-tetraethyl-1,3,2-dioxaborolan-2-yl)phenyl)-1,2,4,5-tetrazine (Tz-Ph-BEpin) (16)**

**
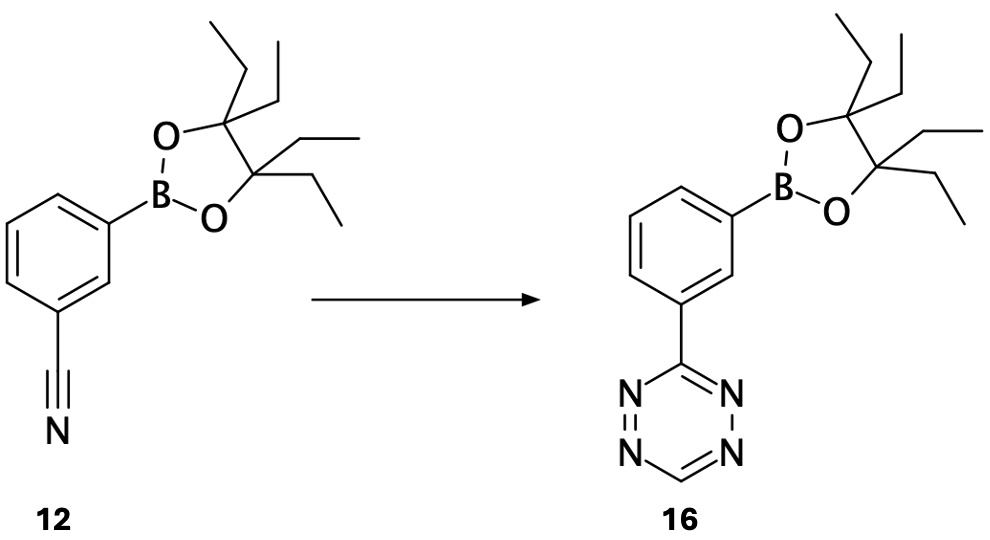
**

In a 15 mL heavy-wall cylindrical Synthware pressure vessel, equipped with a magnetic stirring bar, formamidine acetate (6 eq, 3.28 g, 31.6 mmol) and hydrazine monohydrate (64–65%; 8.5 eq, 2.17 mL, 44.7 mmol) were mixed in 5.2 mL ethanol. The reaction mixture was pipetted up and down multiple times to dissolve the formamidine salt. After adding 3-mercaptopropionic acid (0.3 eq, 137 µL, 1.5 mmol) as the catalyst, CN-Ph-BEpin (**12**; 1 eq, 1.5 g, 5.3 mmol) was added and the closed pressure vessel was heated to 60^o^C in a sand bath under magnetic stirring for 22 hours. The pressure vessel was removed from the sand bath to cool down to room temperature for 30 minutes and then opened gently to release potential overpressure. The reaction mixture was transferred to a 500 mL conical flask, upon which 50 mL DCM and 4.5 g sodium nitrite in 30 mL water were added under magnetic stirring. Oxidation towards the final product was initiated by slowly adding 12 mL glacial acetic acid to reach a pH of 5–5.5 (reaction mixture turns bright red). The reaction mixture was left for 15 minutes before the product was extracted 3 times using DCM and water. The organic phase was dried over magnesium sulphate, filtered and concentrated *in vacuo*. Purified Tz-Ph-BEpin (**16**; 32.7 mg, 2% yield) was obtained *via* manual column chromatography (hexane/ethyl acetate 95/5 (v/v)) as a red solid. TLC Mobile Phase B was used for qualitative analysis. ^1^H NMR (400 ΜΗz, CDCl_3_) *δ* 10.22 (s, 1H), 9.07 (br, 1H), 8.70 ddd (*J* = 7.9, 1.6, 0.40 Hz, 1H), 8.10 (dt, *J* = 7.36, 1.13 Hz, 1H), 7.61 (t, *J* = 7.68 Hz, 1H), 1.89–1.69 (m, 8H), 1.00 (t, *J* = 7.5 Hz, 12H). ^13^C NMR (101 MHz, CDCl_3_) *δ* 171.30 (s, 1C), 166.81 (s, 1C), 157.95 (s, 1C), 139.64 (s, 1C) 134.9 (s, 1C), 131.11 (s, 1C), 130.90 (s, 1C), 128.86 (s, 1C), 89.39 (s, 2C) 26.66 (s, 4C), 9.02 (s, 4C) (Supplementary Figure S6). LC-MS (API-ES) *m/z*: [M + H]^+^ calculated: 340.2; found: 341.

**Synthesis of 3‐(3‐fluorophenyl)‐1,2,4,5‐tetrazine (Tz-Ph-F) (14)**

**
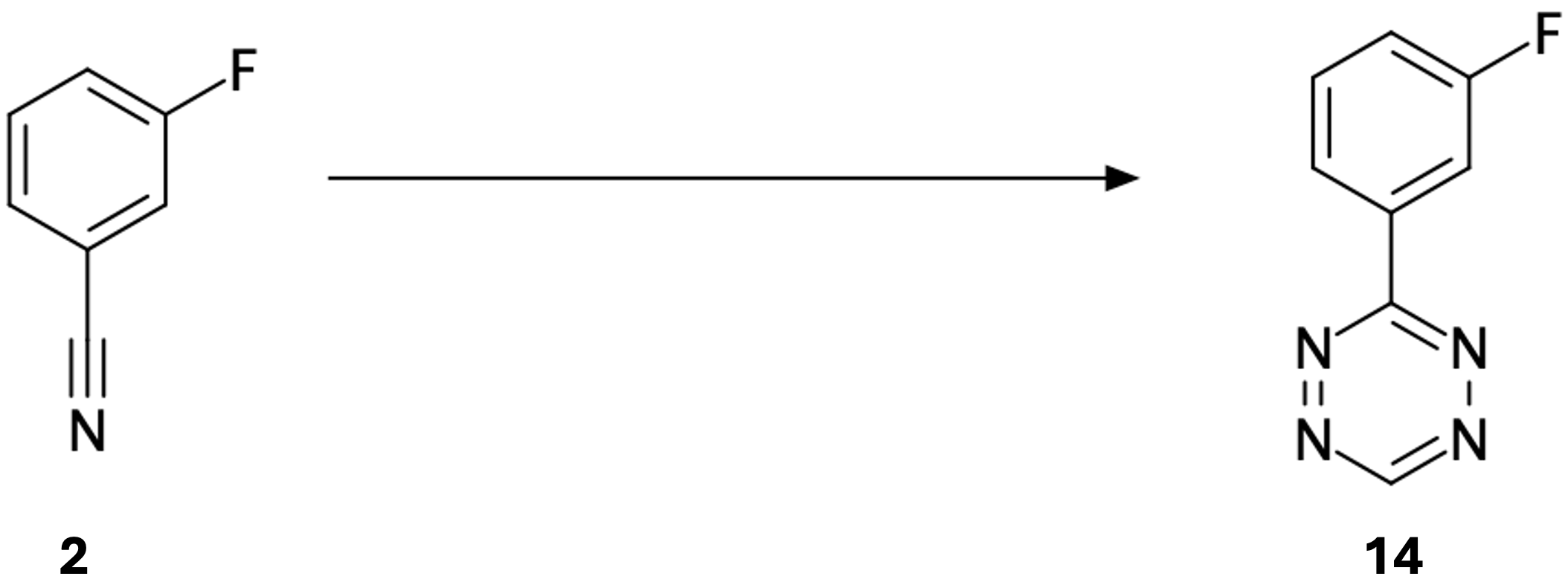
**

In a 15 mL heavy-wall cylindrical Synthware pressure vessel, equipped with a magnetic stirring bar, formamidine acetate (5.0 g, 48 mmol) and hydrazine monohydrate (64–65%; 3.19 mL, 65.6 mmol) were mixed in 8 mL ethanol. The reaction mixture was pipetted up and down multiple times to dissolve the formamidine salt. After adding 3-mercaptopropionic acid (166 µL, 1.6 mmol) as the catalyst, 3-fluorobenzonitrile (**2**; 855 µL, 8.0 mmol) was added and the closed pressure vessel was heated to 60^o^C in a sand bath under magnetic stirring for 22 hours. The pressure vessel was removed from the sand bath to cool down to room temperature for 30 minutes and then opened gently to release potential overpressure. The reaction mixture was transferred to a 500 mL conical flask, upon which 50 mL DCM and 6.0 g sodium nitrite in 15 mL water were added under magnetic stirring. Oxidation towards the final product was initiated by slowly adding 50 mL 1 M HCl to reach a pH of 4 (reaction mixture turns bright red). The reaction mixture was left for 15 minutes before the product was extracted 3 times using DCM and water. The organic phase was dried over magnesium sulphate, filtered and concentrated *in vacuo*. Purified Tz-Ph-F (**14**; 480 µg, 34% yield) was obtained *via* column chromatography (100% toluene) as a red solid. TLC Mobile Phase A was used for qualitative analysis. ^1^H NMR (400 ΜΗz, CDCl_3_) *δ* 10.26 (s, 1H), 8.44 (dt, *J* = 7.8, 1.3 Hz, 1H), 8.33 (ddd, *J* = 9.7, 2.6, 1.6 Hz, 1H) 7.60 (td, *J* = 8.1, 5.7 Hz, 1H) 7.36 (tdd, *J* = 8.3, 2.6, 1.0 Hz, 1H). ^13^C NMR (101 MHz, CDCl_3_) *δ* 165.86 (d, *J* = 3.2 Hz, 1C), 163.17 (d, *J* = 247.65, 1C), 158.18 (s, 1C), 133.90 (d, *J* = 8 Hz, 1C) 131.24 (d, *J* = 8 Hz, 1C), 120.47 (d, *J* = 3.2 Hz, 1C), 120.36 (d, *J* = 21.06 Hz, 1C), 115.32 (d, *J* = 24 Hz, 1C) (Supplementary Figure S7). LC-MS (API-ES) *m/z*: [M + H]^+^ calculated: 176.2; found: 177.

**Synthesis of 4‐cyano‐*N*,*N*,*N*‐trimethylanilinium triflate (*p*-CN-Ph-N^+^Me_3_) (17)**

**
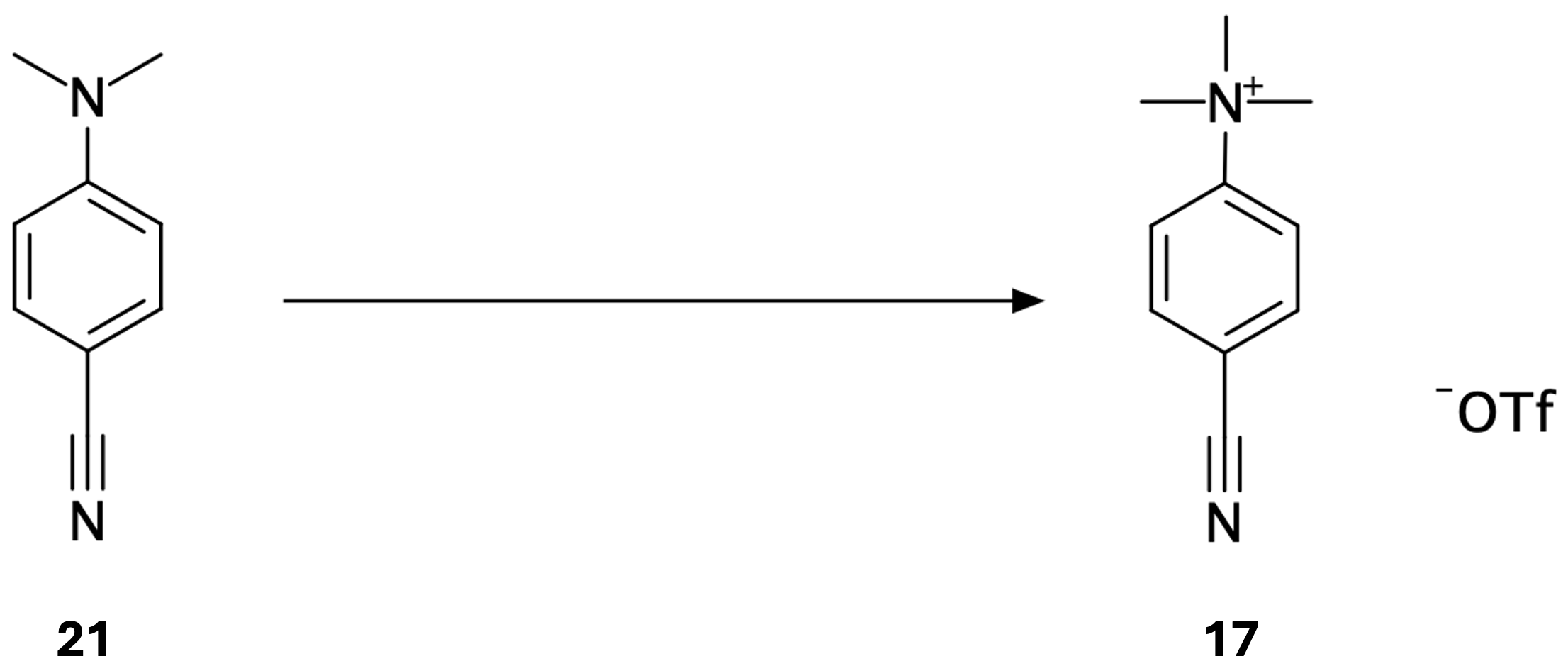
**

In a pre-dried reaction vial, 190 µL methyl trifluoromethanesulfonate (1.1 eq, 1.73 mmol) was added to a solution of 4-(dimethylamino)benzonitrile (**21**; 1 eq, 230 µg, 1.57 mmol) in 2 mL dry DCM. The mixture was left stirring overnight in a closed reaction vial, upon which a crystalline solid was formed. The DCM was gently removed using a Pasteur pipette and the crystalline solid was washed twice with 2 mL DCM. The product was dried for 5 minutes under an air flow, yielding *p*-CN-Ph-N^+^Me_3_  (**17**; 214 µg, 44% yield) as white crystalline solid. ^1^H NMR (400 MHz, D_2_O) *δ* 8.06 (s, 4H), 3.70 (s, 9H). ^13^C-NMR (101 MHz, D_2_O) *δ* 149.66 (s, 1C, Ar**C3**), 134.70 (s, 2C), 121.19 (s, 2C), 117.66 (s, 1C), 113.81 (s, 1C), 56.89 (s, 3C) (Supplementary Figure S8). LC-MS (API-ES) *m/z*: [M + H]^+^ calculated: 161.2; found: 162.

**Evaluation of elution conditions**

WAX 1 cc Vac (30 mg), WAX 3 cc Vac (60 mg) or WAX Plus Short (225 mg) cartridges were prewetted with 5 mL water without the need to introduce any preconditioning anions or additives. Non-carrier-added [^18^F]fluoride in [^18^O]H_2_O (0.5–3 GBq, 0.5–2 mL) was directly trapped on the WAX cartridges. To remove residual water, the cartridge was purged once with a 15 mL syringe filled with air, followed by a wash using 5 mL MeCN and another purge using an air-filled 15 mL syringe. For the 225 mg cartridges, [^18^F]fluoride was loaded and washed via the male side. The [^18^F]fluoride was eluted slowly using organic solvent (DMA, DMF, DMSO, DMI, EtOH, MeCN or NMP), with or without the addition of 3% weak organic base (pyridine, triethylamine (TEA)). Cartridges were eluted with 5 mL in total in fractions of 2× 1 mL and 1× 3 mL, with the majority of activity eluting in the first fraction (Supplementary Table S2). The combined trapping and release efficiency (CTRE) was defined as the ratio of eluted activity relative to the starting activity before trapping on the cartridge. CTRE was reported as the average of n ≥ 3 elutions for the 30 mg and 60 mg cartridges and as the average of n = 2 elutions for the 225 mg cartridge.

**General radiofluorination procedure**

All glassware was pre-dried at 105 °C overnight before use. No-carrier-added [^18^F]fluoride was trapped via the male side on a prewetted (5 mL water) WAX Plus Short (225 mg) cartridge. Residual water was removed by purging the cartridge once via the male side with an air-filled 15 mL syringe, followed by a wash using 5 mL MeCN and another purge with air. The [^18^F]fluoride was eluted slowly using 3% pyridine in DMA in fractions of 2× 1 mL and 1× 3 mL. The fraction with the highest level of radioactivity was used for further reactions, with 10–100 MBq added per reaction. For high molar activity (A_m_) reactions, 0.5–1 GBq was added per reaction. Precursor (2.0 mg) and copper(II)triflate (2 eq.) were added to a pre-dried 4 mL screw-cap vial containing a magnetic stirrer. 3% Pyridine in DMA was added to achieve a total reaction volume of 500 µL. The reaction mixture was stirred in a closed reaction vial for 5 minutes at 140 °C. After letting the reaction mixture cool down for approximately 2 minutes, analytical samples were analysed by iTLC to calculate the radiochemical conversion (RCC: defined as the area-under-curve (AUC) for product divided by the total AUC of any (by)products and free [^18^F]fluoride at the baseline) and by analytical radio-HPLC (Method B) to confirm successful synthesis of the desired radiofluorinated product (Supplementary Figures S9–S14). For tetrazine product 3‐(3‐[^18^F]fluorophenyl)‐1,2,4,5‐tetrazine ([^18^F]**14**), unlabelled ^18^F and other impurities were removed by quenching the reaction mixture with 20 mL water and trapping the product on a Waters Sep-Pak^®^ C18 Plus Short cartridge (conditioned using 5 mL ethanol and then 10 mL water). The product was eluted slowly in 0.5 mL fractions of ethanol. Radiochemical purity (RCP) of the isolated product was confirmed by iTLC. The decay-corrected radiochemical yield (RCY) from end-of-bombardment (EOB) to product isolation was defined as the product of the CTRE and the RCY from start-of-synthesis (SOS) to product isolation (RCY_EOB_ = CTRE × RCY_SOS_). RCC, RCY, RCP and A_m_ were reported as the average of n ≥ 3 radiofluorination reactions. Outliers were defined as measurements lower than 1.5 times the interquartile range (IQR) below the lower quartile (Q_1_) or 1.5 times the IQR above the upper quartile (Q_3_) as per standard practice and were removed from average calculations.

**Optimisation of radiofluorination conditions using CN-Ph-BPin**

The general radiofluorination procedure was followed as described above. Different amounts of 3‐(4,4,5,5‐tetramethyl‐1,3,2‐dioxaborolan‐2‐yl)benzonitrile (CN-Ph-BPin, (**1**)) (0.5, 1.0, 2.0, 5.0 g) and copper(II)triflate (0.5, 1.0, 1.5, 2.0, 2.5, 3.0 eq.) were added to a pre-dried 4 mL screw-cap vial containing a magnetic stirrer. [^18^F]fluoride (20–100 MBq) and 0–3% pyridine in organic solvent (DMA, DMF, NMP) were added to achieve a total reaction volume of 500 µL, after which the reaction mixture was stirred in a closed reaction vial. Different reaction times (5, 15, 30 minutes) and temperatures (100, 120, 140, 160 °C.) were tested. To test the effect of water on the RCC, a small volume of water (1, 5 µL) was added to the reaction mixture. RCC was reported as the average of n ≥ 3 radiofluorination reactions. When one parameter was being tested, the other parameters were kept constant at 2 mg precursor, 2 equivalents of Cu(OTf)_2_ in 3% pyridine in DMA, with a reaction time of 5 minutes at 140 °C.

**Radiofluorination under stronger basic conditions**

The general trapping and elution procedure was followed as described above. The [^18^F]fluoride was eluted slowly using pure DMSO in fractions of 2× 1 mL and 1× 3 mL. The fraction with the highest level of radioactivity was used for further reactions, with 20–100 MBq added per reaction. Precursor (2.0 mg) and TBAHCO_3_ (1 eq.) as the base were added to a pre-dried 4 mL screw-cap vial containing a magnetic stirrer. Pure DMSO was added to achieve a total reaction volume of 500 µL. The reaction mixture was stirred in a closed reaction vial for 30 minutes at 140 °C. After letting the reaction mixture cool down for approximately 2 minutes, analytical samples were analysed by iTLC and analytical radio-HPLC (Method B). RCC was reported as the average of n ≥ 3 radiofluorination reactions.

**SUPPLEMENTARY TABLES**

| *Supplementary Table S1. Combined trapping and release efficiency (CTRE) for different elution solutions.* *Total elution volume: 5 mL. Base concentration: 3%.* | | | | |
| --- | --- | --- | --- | --- |
| **Size (mg)** | **Solvent** | **Base** | **CTRE (%)**  **Average (range)** |  |
| 30 | DMA | pyridine | 86 (85–87) |  |
|  |  | TEA | 88 (85–90) |  |
|  |  | - | 85 (83–88) |  |
| 60 | DMA | pyridine | 80 (76–82) |  |
|  | DMA | - | 78 (72–83) |  |
|  | DMF | - | 81 (80–83) |  |
|  | DMI | - | 76 (74–79) |  |
|  | DMSO | - | 88 (87–89) |  |
|  | EtOH | - | 56 (44–71) |  |
|  | MeCN | - | 7 (7–8) |  |
|  |  | TEA | 79 (77–83) |  |
| 225 | DMA | pyridine | 90 (89–91) |  |
|  | DMF | pyridine | 81 (80–81) |  |
|  | DMI | pyridine | 83 (81–85) |  |
|  | NMP | pyridine | 87 (86–87) |  |
|  | DMSO | - | 86 (85–87) |  |

| *Supplementary Table S2. Fractionated elution from the WAX cartridges, illustrating the amount of eluted activity (% from total eluted) in each fraction.* | | | | |
| --- | --- | --- | --- | --- |
| **Solvent** | **Cartridge size (mg)** | **Fraction 1** | **Fraction 2** | **Fraction 3** |
|  |  | **1 mL** | **1 mL** | **3 mL** |
| DMA + pyridine | 30 | 92%  (91–92%) | 5%  (4–6%) | 3%  (2–4%) |
|  | 225 | 84%  (82–85%) | 13%  (11–14%) | 3%  (3–3%) |
| DMF + pyridine | 225 | 48%  (48–49%) | 32%  (31–32%) | 20%  (20–20%) |
| DMI + pyridine | 225 | 52%  (40–64%) | 33%  (21–46%) | 15%  (14–15%) |
| NMP + pyridine | 225 | 83%  (83–83%) | 13%  (13–13%) | 5%  (5–5%) |
| DMSO | 225 | 68%  (66–70%) | 25%  (24–26%) | 7%  (6–8%) |

**SUPPLEMENTARY FIGURES**

| **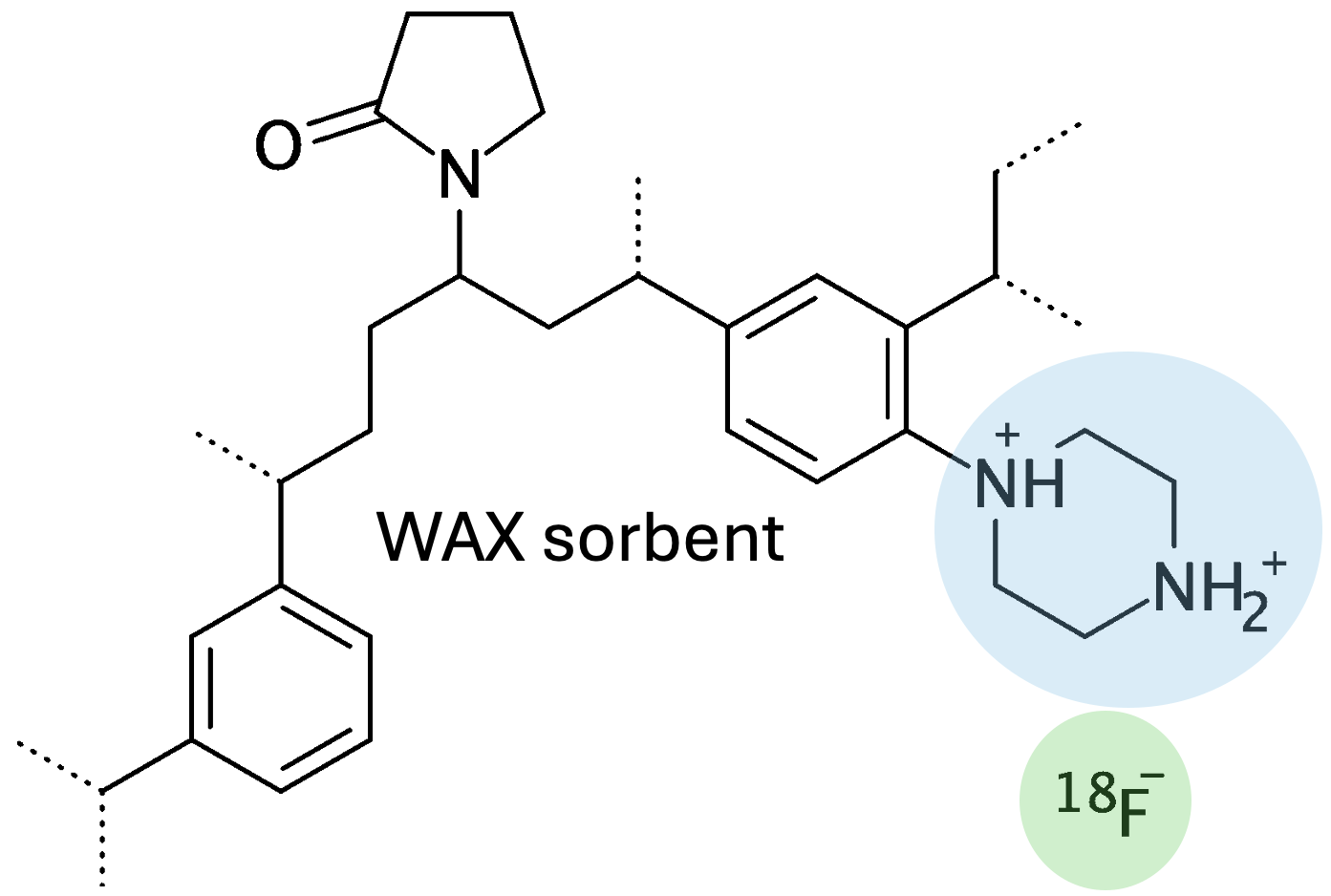** |
| --- |
| *Supplementary Figure S1. Weak anion-exchange interaction mechanism between [^18^F]fluoride and the piperazine-based WAX cartridge.* |

| **A** | **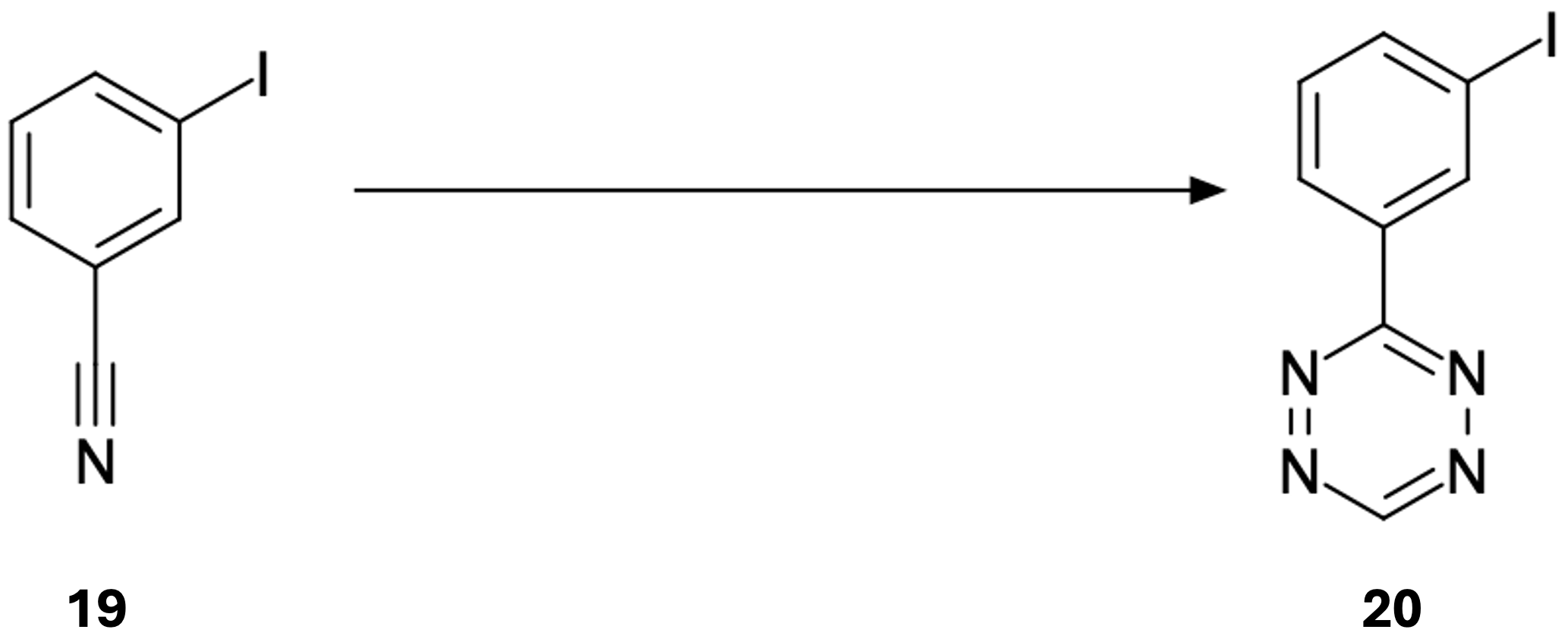**  *  * |
| --- | --- |
| **B** | **** |
| *Supplementary Figure S2. A) ^1^H NMR and B) ^13^C NMR spectra of* ***20****. * Solvent peak (CDCl_3_)* | |

| **A** | **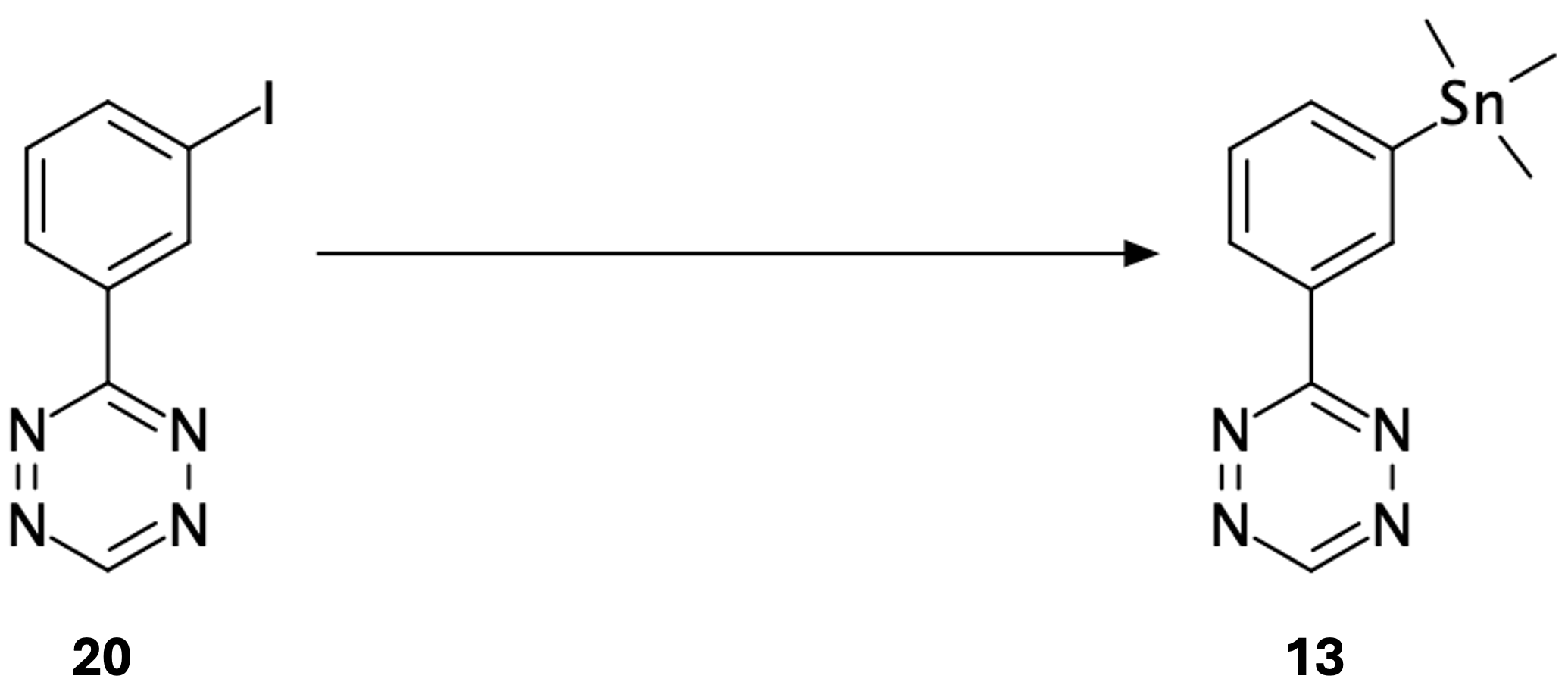**  * |
| --- | --- |
| **B** | ****  * |
| *Supplementary Figure S3. A) ^1^H NMR and B) ^13^C NMR spectra of* ***13****. * Solvent peak (CDCl_3_)* | |

| **A** | **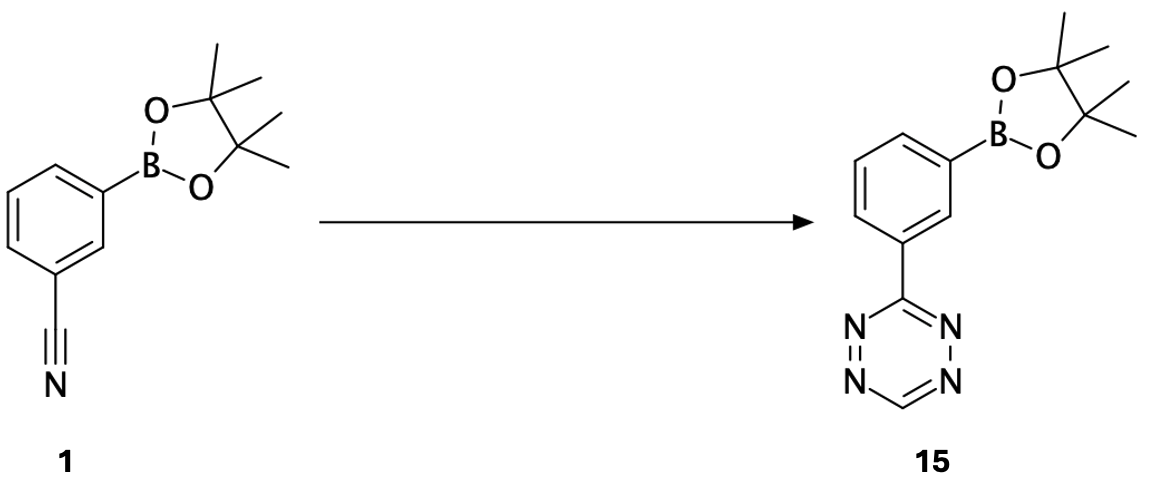**  H_2_O  * |
| --- | --- |
| **B** | ****  * |
| *Supplementary Figure S4. A) ^1^H NMR and B) ^13^C NMR spectra of* ***15****. * Solvent peak (CDCl_3_)* | |

| **A** | **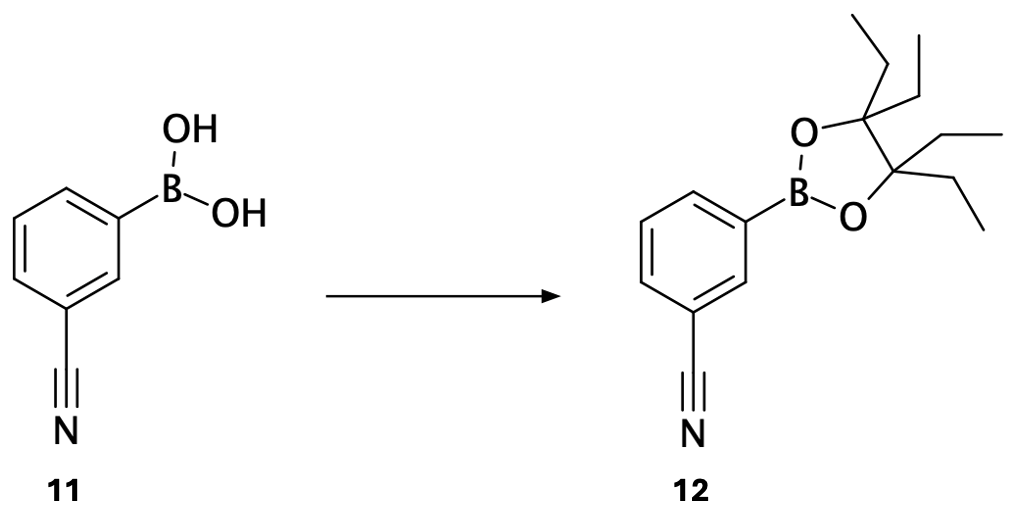**  * |
| --- | --- |
| **B** | ****  * |
| *Supplementary Figure S5. A) ^1^H NMR and B) ^13^C NMR spectra of* ***12****. * Solvent peak (CDCl_3_)* | |

| **A** | **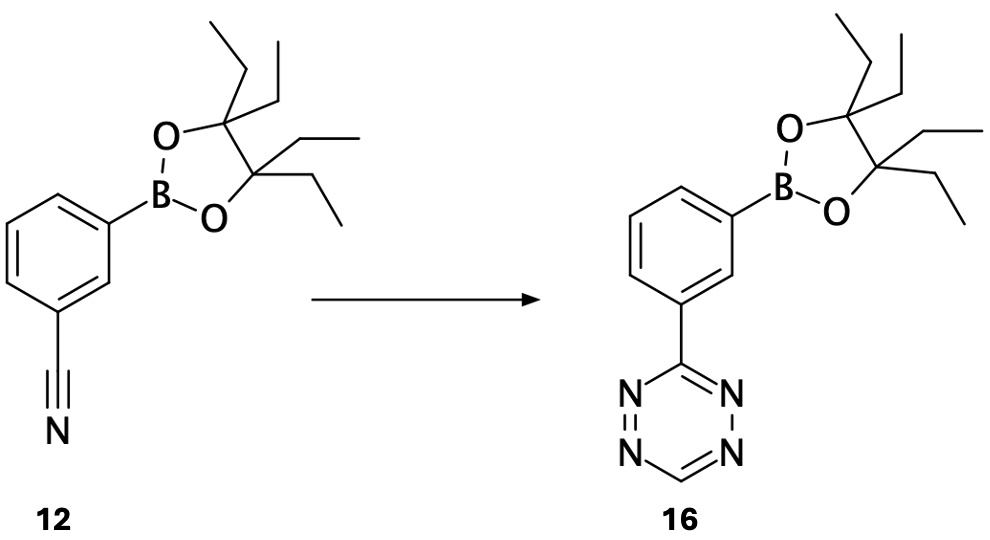** ****  H_2_O  *  * |
| --- | --- |
| **B** | **** |
| *Supplementary Figure S6. A) ^1^H NMR and B) ^13^C NMR spectra of* ***16****. * Solvent peak (CDCl_3_).* | |

| **A** | **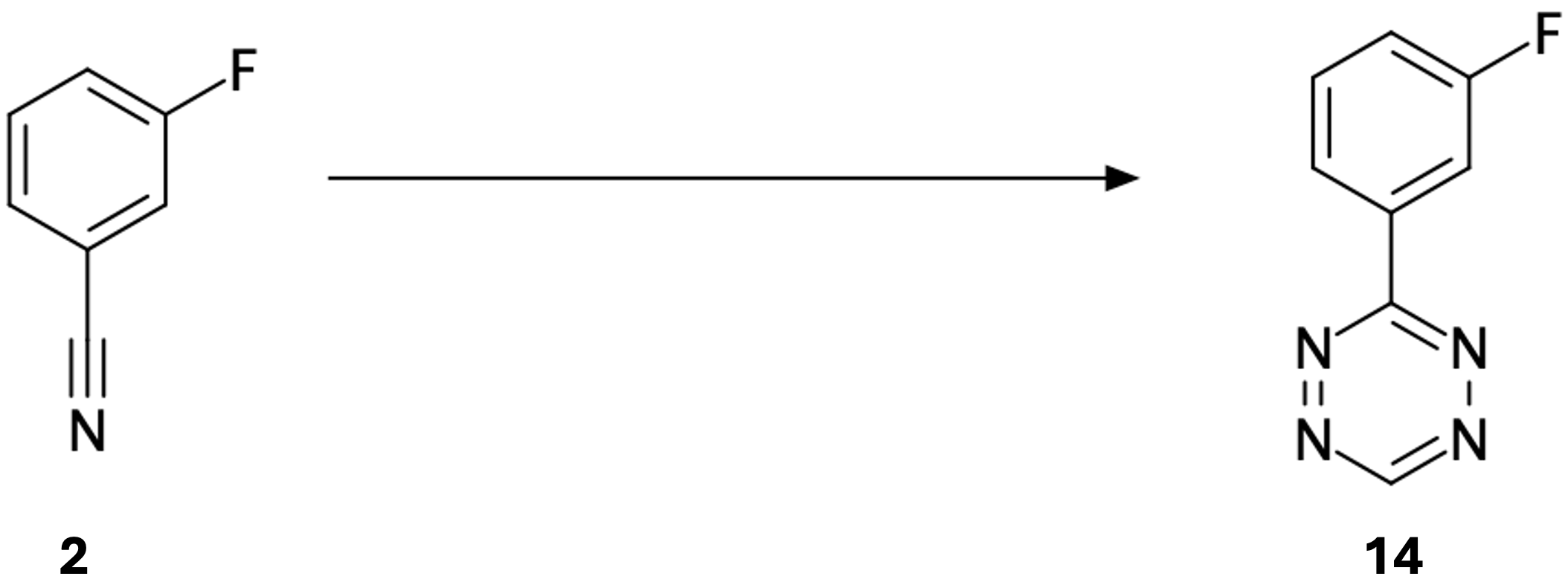**  *  grease  grease  H_2_O  * |
| --- | --- |
| **B** | **** |
| *Supplementary Figure S7. A) ^1^H NMR and B) ^13^C NMR spectra of* ***14****. * Solvent peak (CDCl_3_)* | |

| **A** | **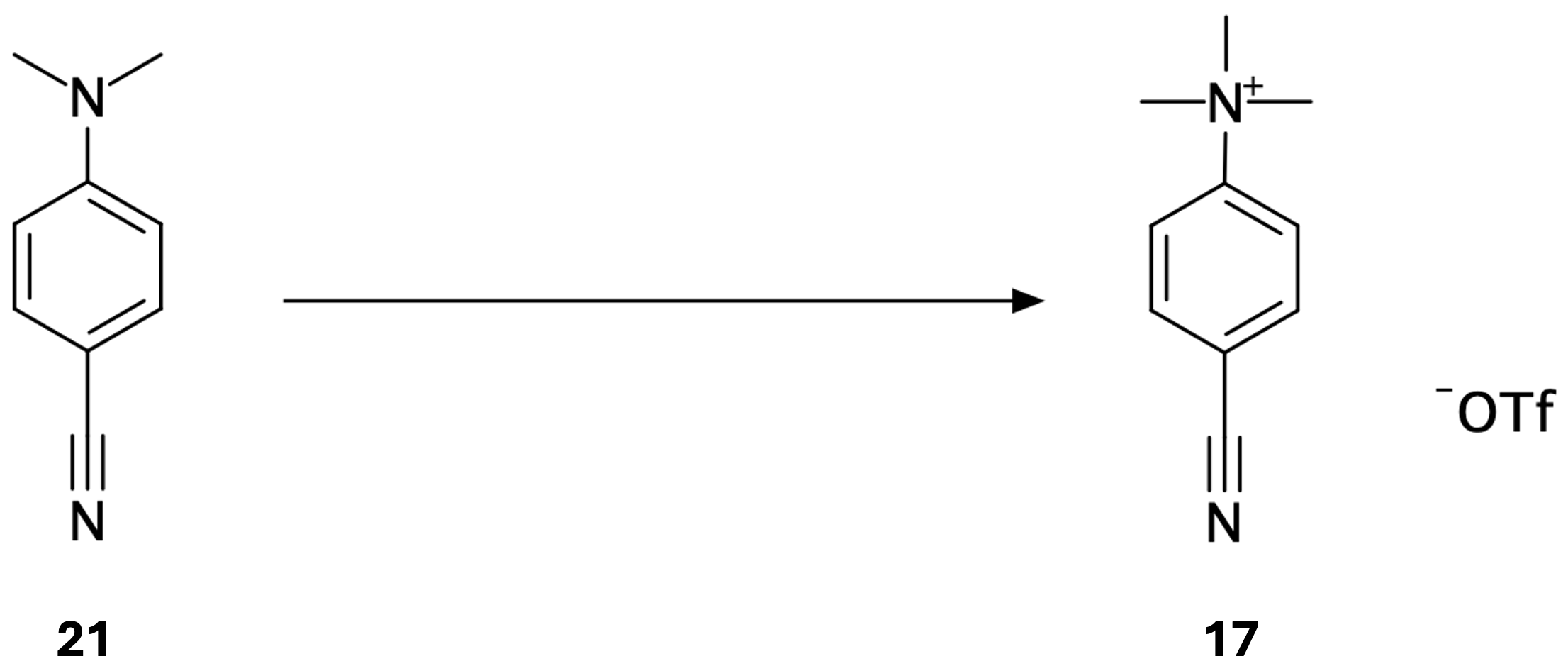**  * |
| --- | --- |
| **B** | **** |
| *Supplementary Figure S8. A) ^1^H NMR and B) ^13^C NMR spectra of* ***17****. * Solvent peak (D_2_O)* | |

| **A** | *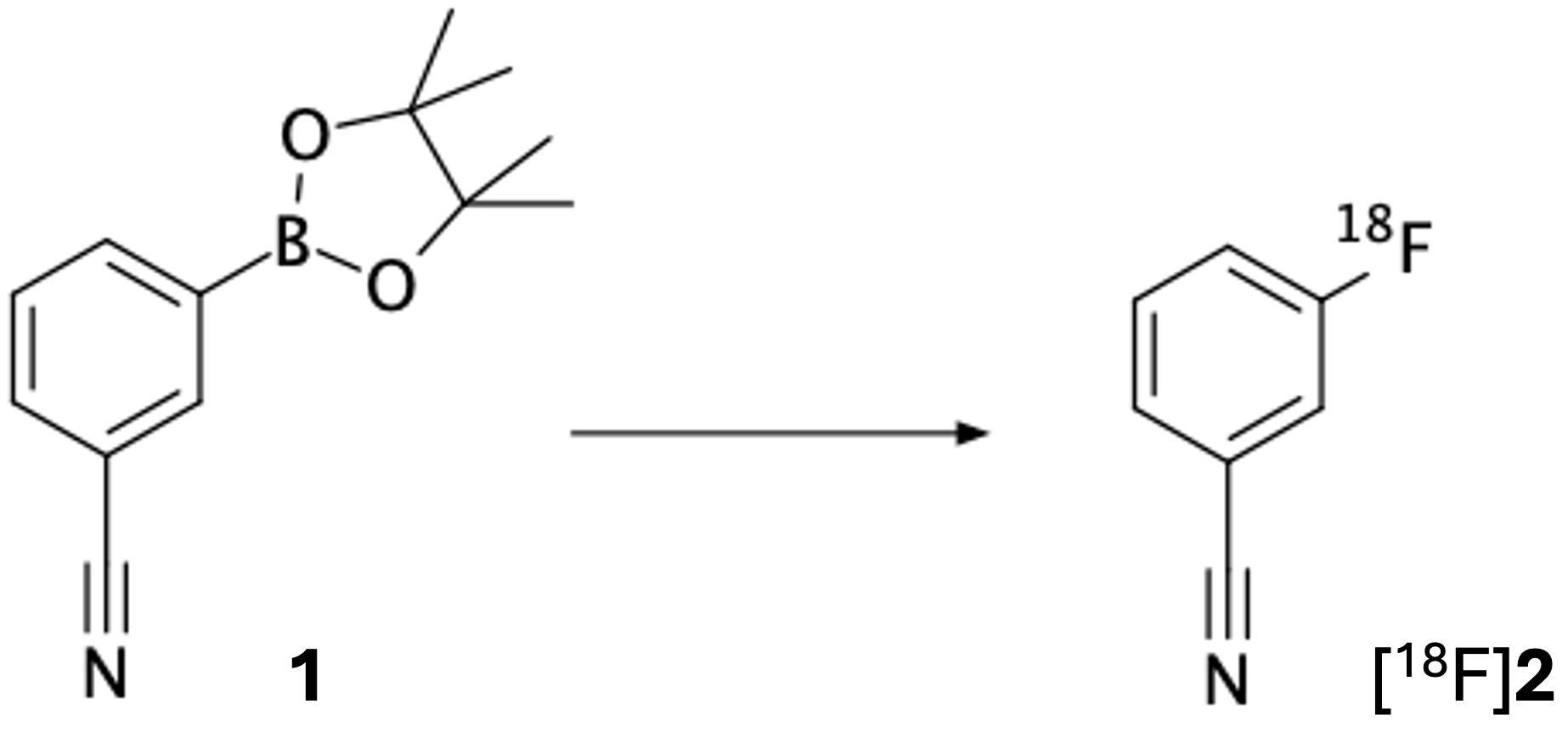* |
| --- | --- |
| **B** | [^18^F]**2**  ^18^F |
| *Supplementary Figure S9. A) Crude radio-HPLC, confirming the successful synthesis of [^18^F]****2****. Top: UV signal of reference compound. Bottom: radioactive signal of [^18^F]****2****. B) Representative iTLC of crude [^18^F]****2****, used to calculate the RCC.* | |

| **A** | *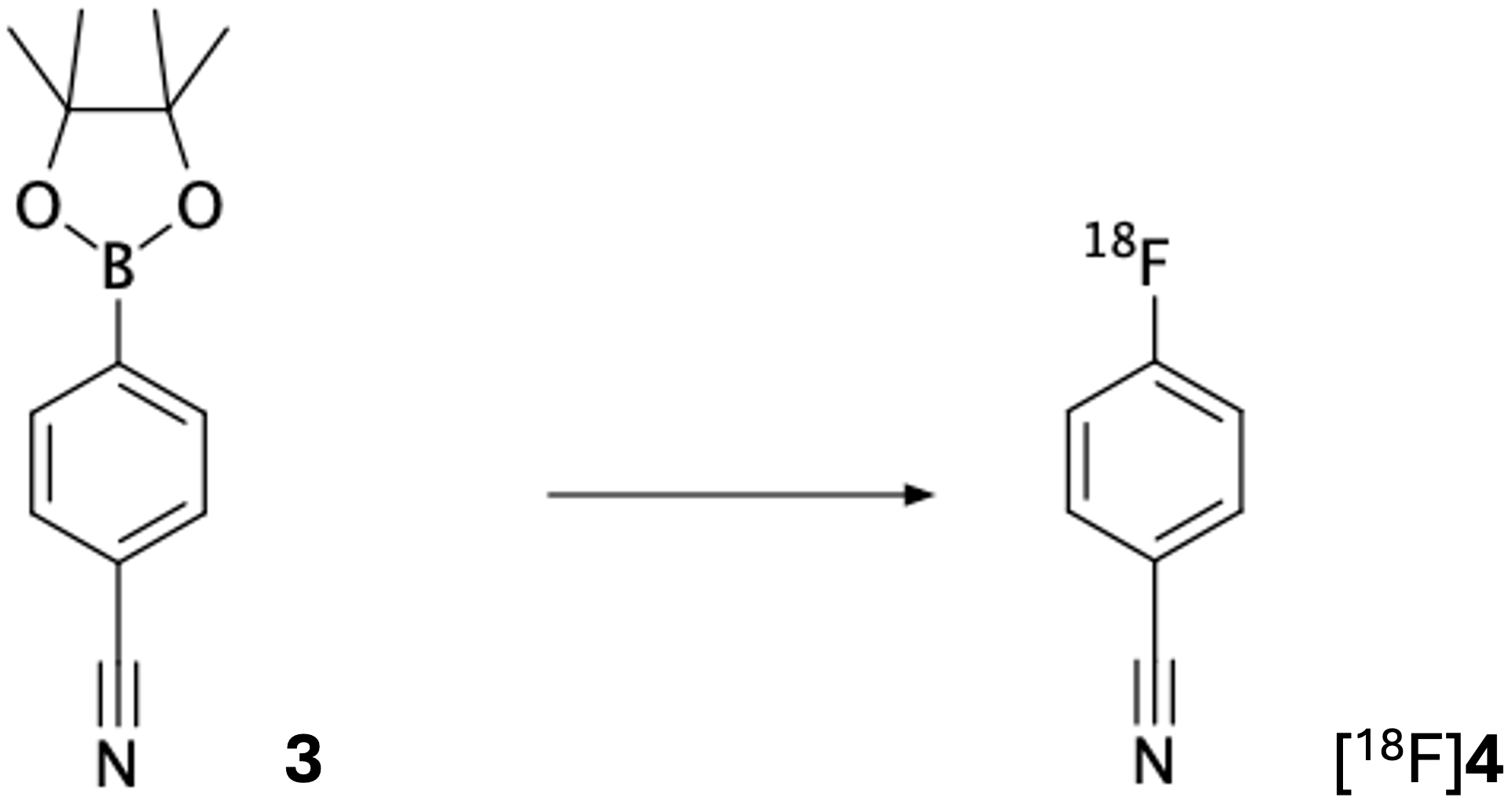* |
| --- | --- |
| **B** | [^18^F]**4**  ^18^F |
| *Supplementary Figure S10. A) Crude radio-HPLC, confirming the successful synthesis of [^18^F]****4****. Top: UV signal of reference compound. Bottom: radioactive signal of [^18^F]****4****. B) Representative iTLC of crude [^18^F]****4****, used to calculate the RCC.* | |

| **A** | *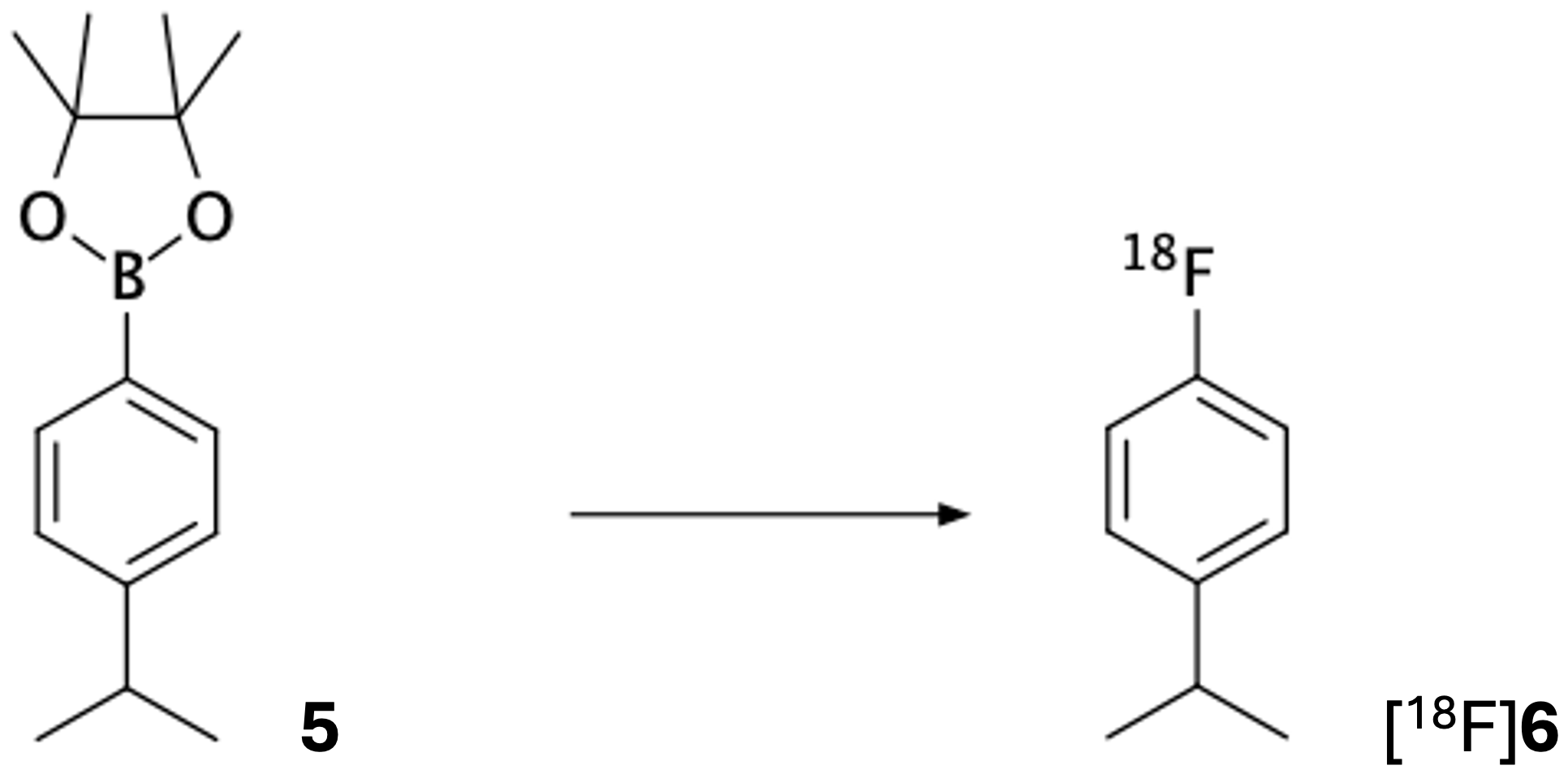* |
| --- | --- |
| **B** | [^18^F]**6**  ^18^F |
| *Supplementary Figure S11. A) Crude radio-HPLC, confirming the successful synthesis of [^18^F]****6****. Top: UV signal of reference compound. Bottom: radioactive signal of [^18^F]****6****. B) Representative iTLC of crude [^18^F]****6****, used to calculate the RCC.* | |

| **A** | *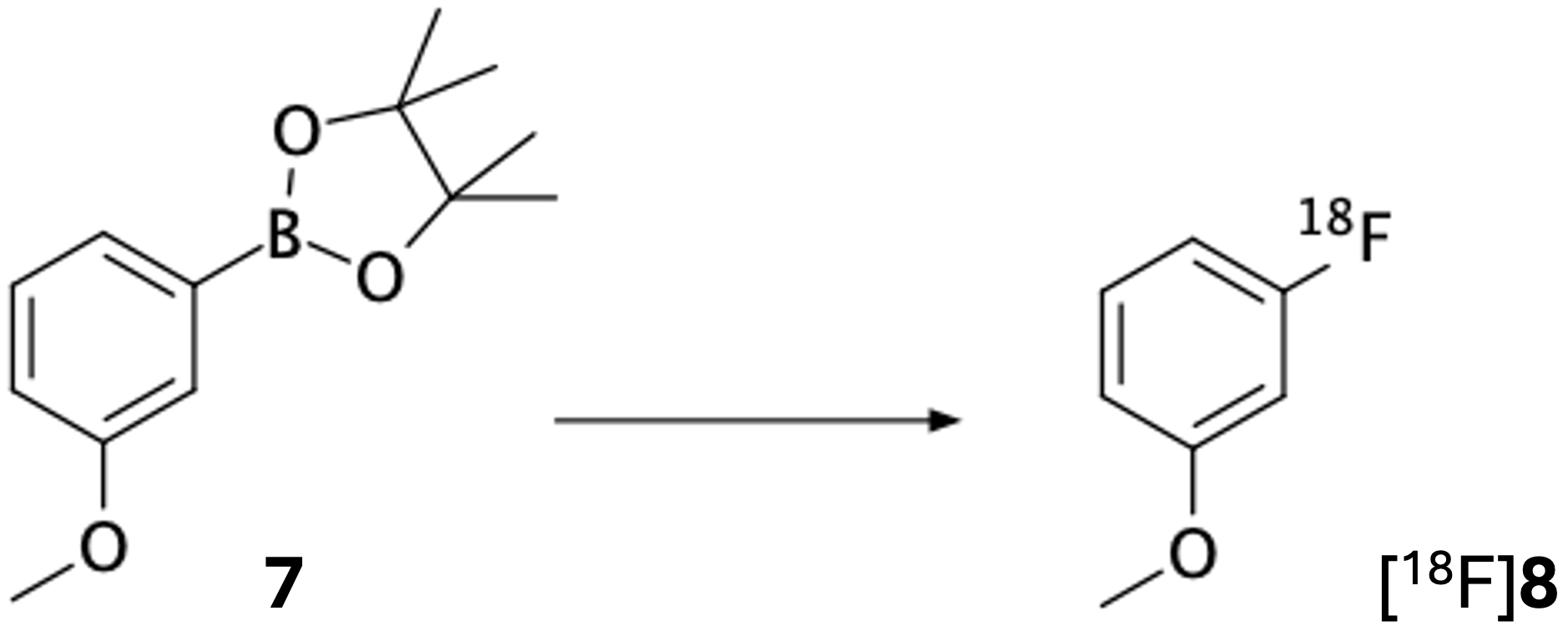* |
| --- | --- |
| **B** | [^18^F]**8**  ^18^F |
| *Supplementary Figure S12. A) Crude radio-HPLC, confirming the successful synthesis of [^18^F]****8****. Top: UV signal of reference compound. Bottom: radioactive signal of [^18^F]****8****. B) Representative iTLC of crude [^18^F]****8****, used to calculate the RCC.* | |

| **A** | *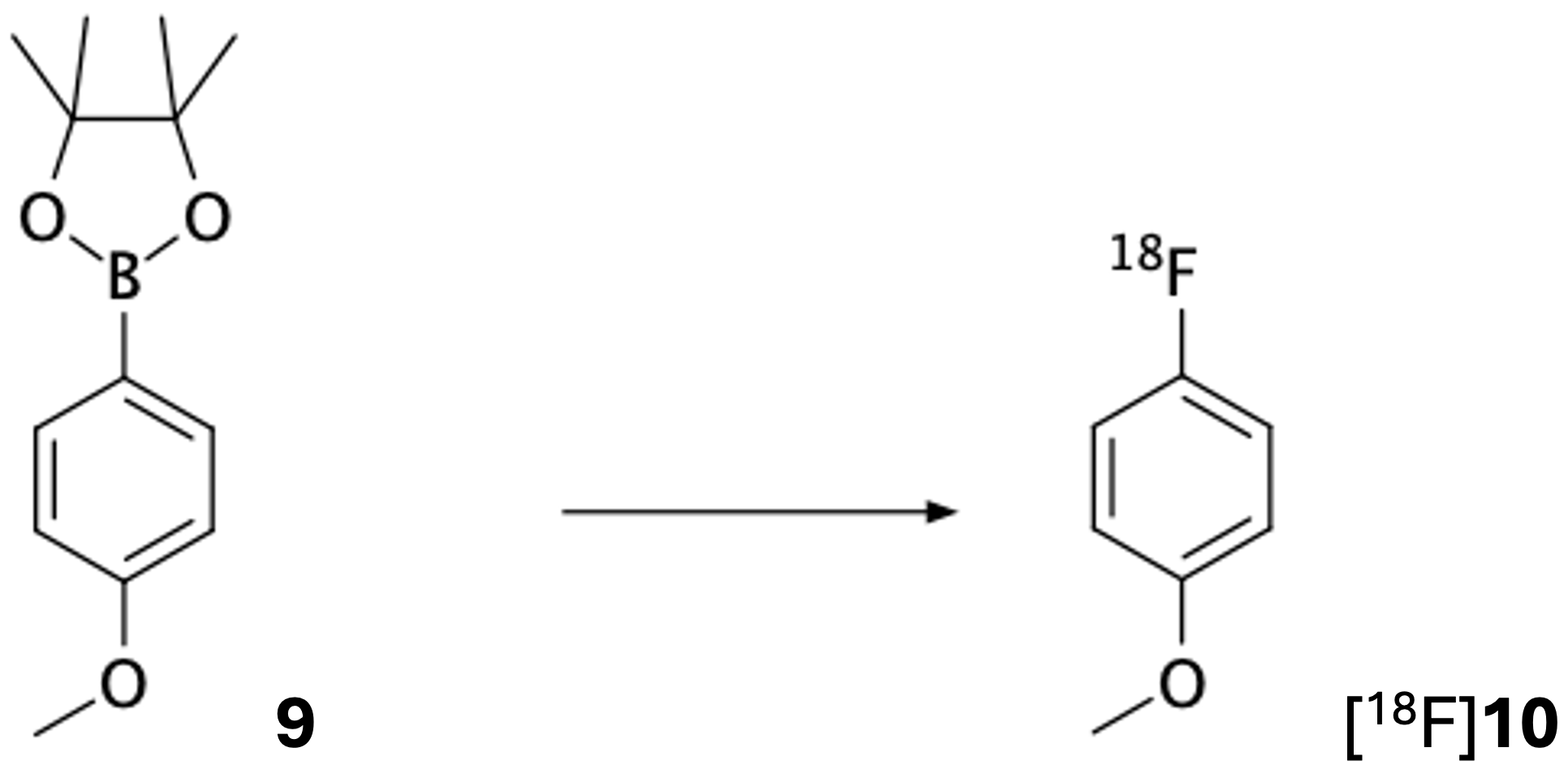*  [^18^F]**10** |
| --- | --- |
| **B** | ^18^F |
| *Supplementary Figure S13. A) Crude radio-HPLC, confirming the successful synthesis of [^18^F]****10****. Top: UV signal of reference compound. Bottom: radioactive signal of [^18^F]****10****. B) Representative iTLC of crude [^18^F]****10****, used to calculate the RCC.* | |

| **A** | 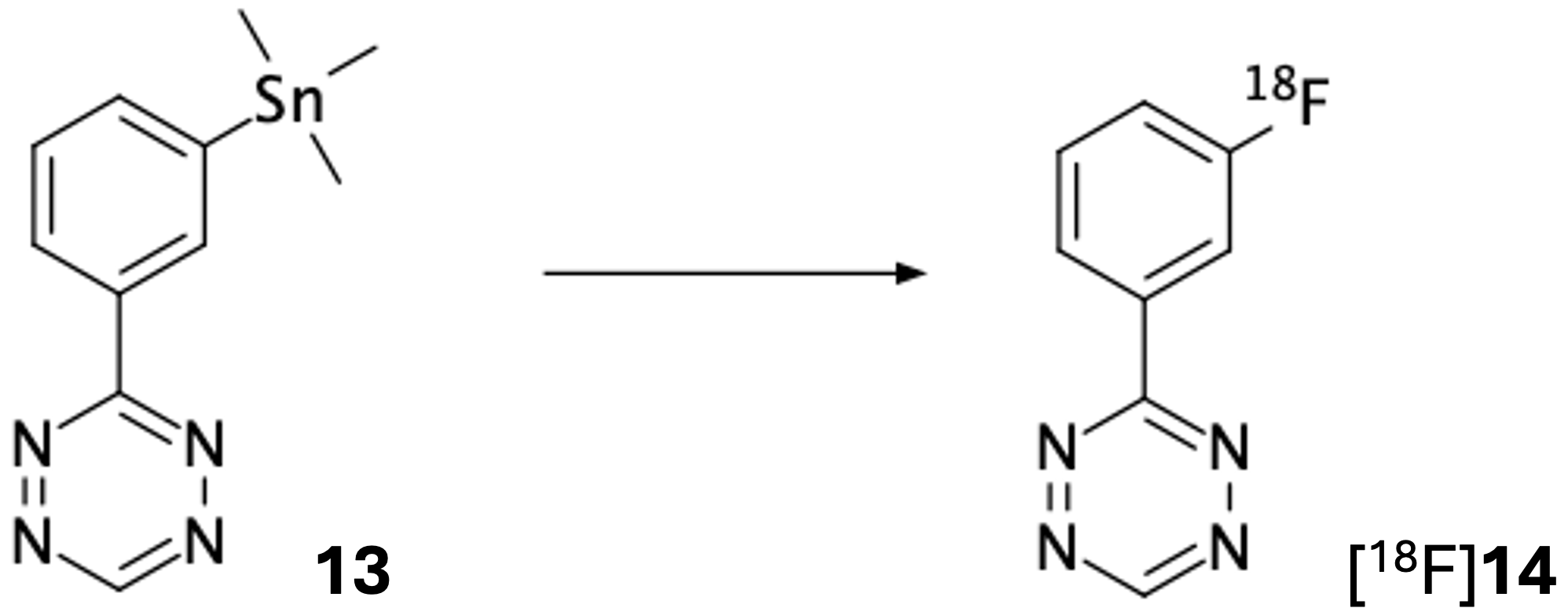 |
| --- | --- |
| **B** | [^18^F]**14**  [^18^F]**14**  ^18^F |
| *Supplementary Figure S14. A) Radio-HPLC of purified tetrazine [^18^F]****14****. Top: UV signal of reference compound. Bottom: radioactive signal of [^18^F]****14****. B) Representative iTLC of crude [^18^F]****14****, used to calculate the RCC (left), as well as purified [^18^F]****14*** *(right).* | |
